# The antigen-presenting molecule MR1 binds host-generated riboflavin catabolites

**DOI:** 10.1084/jem.20250711

**Published:** 2025-11-26

**Authors:** Mohamed R. Abdelaal, Jieru Deng, Mitchell P. McInerney, Emi Ito, Anthony W. Purcell, Sho Yamasaki, Jose A. Villadangos, Hamish E.G. McWilliam, Nicholas A. Gherardin, Jamie Rossjohn, Wael Awad

**Affiliations:** 1 Infection and Immunity Program and Department of Biochemistry and Molecular Biology, Biomedicine Discovery Institute, Monash University, Clayton, Australia; 2 Department of Microbiology and Immunology, Peter Doherty Institute for Infection and Immunity, University of Melbourne, Melbourne, Australia; 3 Department of Molecular Immunology, Research Institute for Microbial Diseases, Osaka University, Suita, Japan; 4 Laboratory of Molecular Immunology, Immunology Frontier Research Centre, Osaka University, Suita, Japan; 5 Institute of Infection and Immunity, Cardiff University School of Medicine, Cardiff, UK; 6 Department of Biochemistry and Pharmacology, Bio21 Molecular Science and Biotechnology Institute, University of Melbourne, Parkville, Australia.

## Abstract

MHC class I–related protein (MR1) presents vitamin B–based antigens (Ags) to mucosal-associated invariant T (MAIT) cells. While microbial riboflavin (RF) precursors are well-documented MR1 ligands, it is unclear whether host-generated RF catabolites influence MR1 immunity. Here, we report that RF catabolites, including 10-formylmethylflavin (FMF), lumichrome, lumiflavin, and alloxazine, bind to MR1 with moderate affinity, while RF itself binds weakly. In contrast to the MR1-upregulating microbial RF precursors, RF catabolites reduced the surface level of MR1 by inducing its retention in the endoplasmic reticulum and inhibiting exit. These RF catabolites weakly competed with vitamin B–based Ags for MR1 binding, thereby selectively inhibiting MAIT activation. The crystal structures of MR1 with RF, FMF, lumiflavin, and lumichrome show binding in the A′-pocket of MR1. Here, lumichrome formed a “flavin bond” covalent interaction with MR1-Lys43 differing from the typical Schiff base. Collectively, we identified three-ringed isoalloxazines that bind MR1 and reduce surface levels, suggesting a potential role in dampening MAIT cell immunity.

## Teaser

This study reveals that host-generated riboflavin breakdown products, including lumichrome and lumiflavin, can bind to the immune protein MR1. Unlike microbial vitamin B precursors, these compounds reduce MR1 surface levels and may naturally dampen MAIT cell immune activation.

## Introduction

The major histocompatibility complex class I–related (MR1) protein captures and presents small organic molecules to an innate-like T cell population called mucosal-associated invariant T (MAIT) cells (Awad et al., 2023; Corbett et al., 2014; Kjer-Nielsen et al., 2012). MAIT cells play crucial roles in both protective and aberrant immunity, as well as mucosal homeostasis (Corbett et al., 2020; Provine and Klenerman, 2020). MR1 is ubiquitously expressed in all cells, but its cell surface expression is dependent on ligand availability. The MR1 ligand-binding A′-pocket has adequate malleability to capture a broad range of chemical structures including vitamin B antigens (VitBAg) exemplified by microbial derivatives of vitamin B2 (riboflavin, RF) precursors that are formed during infection with a wide range of RF-producing microbes (Corbett et al., 2014). The range of MR1 ligands, however, extends to other VitBAgs including vitamin B9 derivatives (6-formylpterin [6-FP] and acetyl-6-FP [Ac-6-FP]) (Eckle et al., 2014; Kjer-Nielsen et al., 2012) and vitamin B6 derivatives (pyridoxal and pyridoxal-5′-phosphate) (McInerney et al., 2024), as well as non-VitBAg compounds, like environmental ligands (e.g., components of cigarette smoke [Awad et al., 2025b]), diet, drugs, and drug-like molecules (Keller et al., 2017; Salio et al., 2020; Wang et al., 2022a). More recently, host-derived nucleobase adducts and sulfated bile acids have been described (Chancellor et al., 2025; Ito et al., 2024; Vacchini et al., 2024). In the setting of RF-derived metabolites, the microbial intermediate 5-amino-6-D-ribitylaminouracil (5-A-RU) nonenzymatically reacts with small glycolysis metabolites (e.g., methylglyoxal) to generate short-lived intermediates, e.g., 5-(2-oxopropylideneamino)-6-D-ribitylaminouracil (5-OP-RU), that are captured by MR1 before conversion to lumazines, e.g., RL-6-Me-7-OH and RL-7-Me, and are potent antigens (Ags) for MAIT cells. These RF-based Ags function as a “microbial metabolite signature” that activates MAIT cells, where 5-OP-RU represents the most potent MAIT cell agonist identified to date (Awad et al., 2020a; Awad et al., 2020b). However, no host-derived RF-related catabolites have been described.

Typically, the binding of MR1 to ligands within the endoplasmic reticulum (ER) of antigen-presenting cells (APCs) triggers ER-resident MR1 to translocate to the cell surface (McWilliam et al., 2016; McWilliam et al., 2020). MR1–ligand complexes remain at the cell surface for several hours before being re-internalized back to the cell interior and/or endosomes (McWilliam et al., 2016), with the majority of MR1 molecules subsequently being degraded, and a minor fraction recycling from endosomes back to the cell surface, potentially loaded with a different ligand. The release of MR1 from the ER to the cell surface involves a conformational change driven by a molecular switch involving a lysine residue (Lys-43) at the base of the ligand-binding groove. Here, the potent MR1-binding ligands form a covalent bond, termed a “Schiff base” with Lys-43, neutralizing its positive charge and stabilizing MR1 (McWilliam and Villadangos, 2024). Indeed, mutation of MR1 Lys-43 to Ala (termed MR1^K43A^) prevents Schiff base formation with these ligands, dramatically reducing Ag presentation (McWilliam et al., 2016; Reantragoon et al., 2013). Notably, some synthetic ligands, such as DB28 and NV18.1 compounds that bind MR1 but do not form Schiff base bonds, can retain MR1 within the ER, leading to a downregulation at the cell surface (Salio et al., 2020), but whether naturally occurring ligands can suppress MR1 upregulation remains unclear.

A number of reports have provided evidence that RF itself cannot activate MAIT cells, but rather blocks their response to bacterial Ag presentation, reminiscent of other nonstimulatory MR1-binding ligands such as 6-FP (Harriff et al., 2018; Kjer-Nielsen et al., 2012; Shibata et al., 2022). The mechanisms of RF-based MAIT cell inhibition, however, are unknown. When RF is catabolized (enzymatically or by photodegradation) in humans, distinct products are formed including the following: 10-formylmethylflavin (FMF), lumiflavin, lumichrome (7,8-dimethylalloxazine), and/or alloxazine (Bitsch and Bitsch, 2016), all of which can be detected in human blood (Barupal and Fiehn, 2019; Hardwick et al., 2004). Given that these RF catabolites are structurally similar to the microbial MR1-binding Ags, we reasoned that MR1 may bind these host RF catabolites. Through cellular, biochemical, and structural approaches, we show that RF catabolites bind MR1 and impact its cell surface levels, thereby showing how host-derived three-ringed compounds can modulate the MR1–MAIT cell axis.

## Results

### RF and RF catabolites bind MR1 molecules

**Figure 1.**
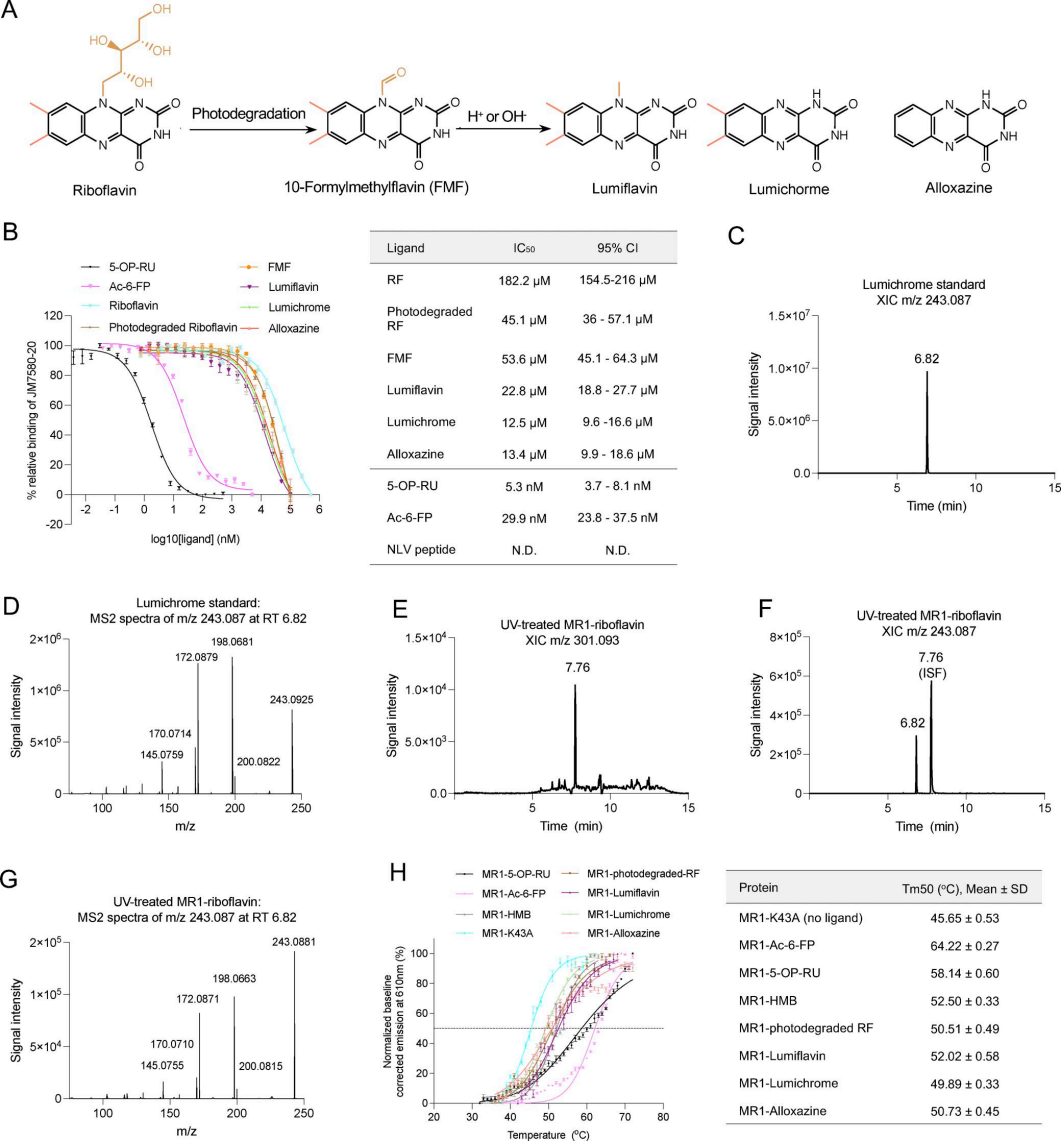
**RF and RF catabolites bind MR1. (A)** Mechanism of metabolism and photodegradation of RF. **(B)** Titration curves of the shown ligands binding to MR1 were obtained from the fluorescence polarization–based assay (left). Each data point represents normalized percentage binding from three independent experiments performed in triplicate. Mean values are plotted with SEM represented in error bars. Curve fit for ligands is displayed in the table (right). **(C and D)** (C) XICs for m/z 243.087 in a lumichrome solvent standard and (D) MS2 fragmentation of the main peak at retention time (RT) 6.82 are depicted. **(E)** UV-treated MR1 RF XIC for m/z 301.0927. **(F)** XIC for m/z 243.087 showing lumichrome present at RT 6.82, with an additional peak at RT 7.76. **(G)** MS2 fragmentation of main peak at RT 6.82, showing aligned fingerprint comparison with lumichrome solvent standard. **(H)** Thermostability of soluble WT MR1 refolded with the indicated ligands was measured by fluorescence-based thermal shift assay. The graph shows baseline-corrected, normalized emission at 610 nm plotted against temperature (°C). Each point represents the mean of three technical replicates, and error bars represent SD. The Tm50 is indicated by the dotted line at 50%. The table on the right shows the mean Tm50 from three independent experiments, each measured in at least a technical triplicate. XICs, extracted ion chromatograms.

To investigate whether RF and its catabolites (Fig. 1 A) are potential ligands for MR1, we quantified the relative binding affinities of these compounds for MR1 using a fluorescence polarization–based cell-free assay (Wang et al., 2022a). The IC_50_ concentrations were interpolated from the non-linear regression of the dose–response curves for MR1–ligand interactions (Fig. 1 B). Consistent with previous reports (Wang et al., 2022a), 5-OP-RU and Ac-6-FP, which form a Schiff base interaction with MR1, show strong binding to MR1 with IC_50_ values 5.3 and 29.9 nM, respectively. The fluorescence polarization assay showed that RF is a weak binder to MR1 with an IC_50_ value of ∼182 µM, consistent with the previous finding that RF can stabilize MR1 on the cell surface of APCs (Shibata et al., 2022). However, when RF was exposed to UV light (30 min) or daylight (6–8 h) (referred to as photodegraded RF hereafter), the IC_50_ reduced to ∼45 µM, suggesting the formation of higher affinity MR1 ligands in solution during RF photodegradation, reminiscent of folic acid photodegradation products having MR1-binding capacity (Kjer-Nielsen et al., 2012). We then measured the IC_50_ for individual RF catabolites (Fig. 1 B), which were moderate binders with IC_50_ values in the range 12–54 µM. Mass spectrometry analysis showed that MR1–photodegraded RF contained a mixture of compounds including lumichrome (MH+ as m/z 243.087) and carboxymethylflavin (MH+ as m/z 301.093) (Fig. 1, C–G).

**Figure S1.**
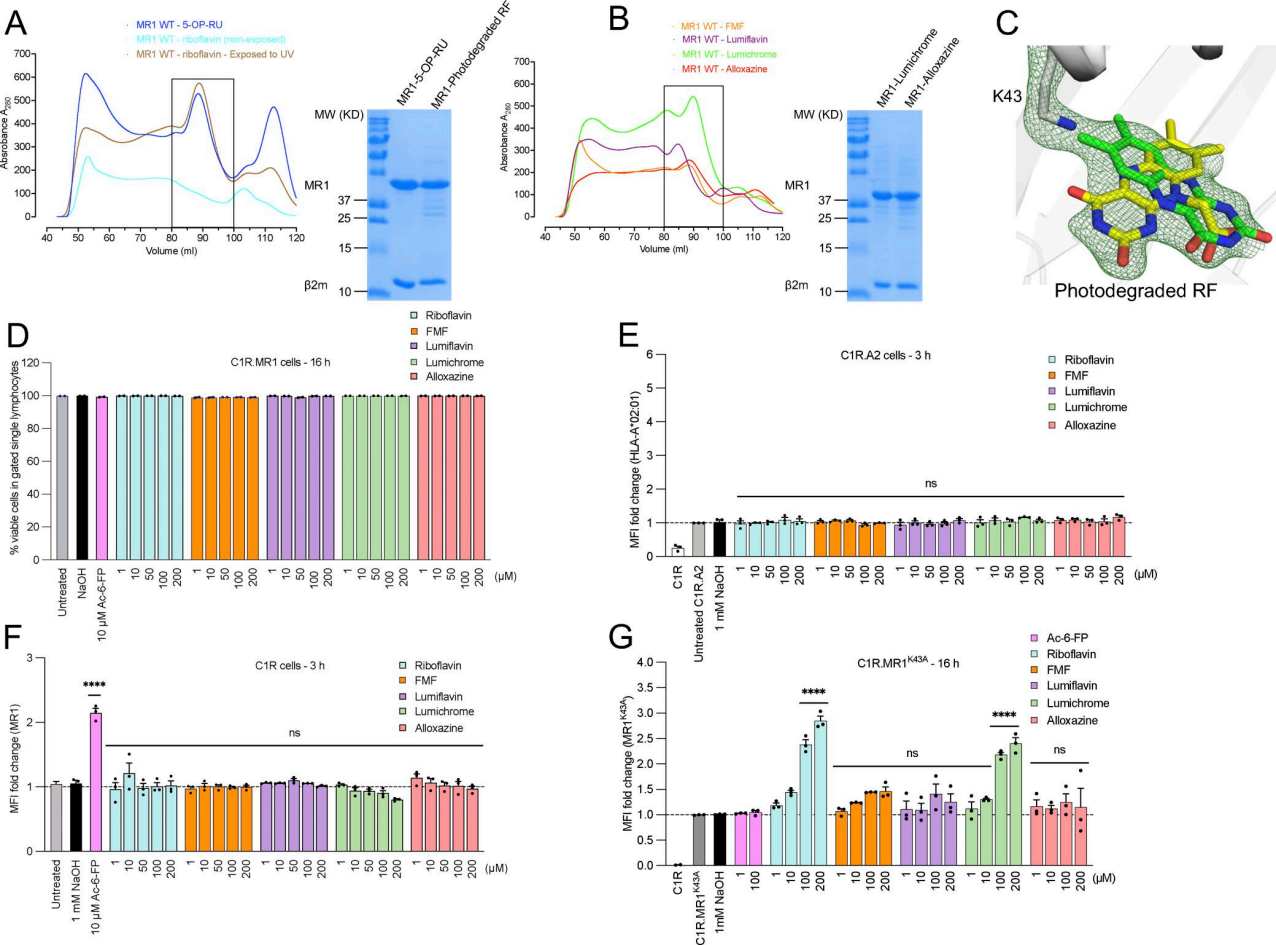
**RF catabolites refold with MR1 and modulate cell surface expression. (A and B)** Shown is the gel filtration (S200 10/300 GL; GE Healthcare) purification (left) and SDS-PAGE analysis (right) of MR1-β2m binary complexes loaded with (A) 5-OP-RU, non-exposed RF, and photodegraded RF, as well as the RF catabolites (B) FMF, lumichrome, lumiflavin, and alloxazine. Absorption at 280 nm and volume (ml) are shown on the y and x axis, respectively. **(C)** Crystallographic omit maps of UV-treated RF are presented as a F_observed_ – F_calculated_ omit map (green mesh) contoured at 2σ showing the position of the RF degradation product(s) within MR1 cleft connected with Lys-43. The superimposed green and yellow sticks represent lumichrome and carboxymethylflavin, respectively, which have been suggested by mass spectrometry to be the main RF photodegradation catabolites in the refold sample. **(D)** Bar graph shows the frequency of viable cells in single lymphocytes gated for C1R.MR1 cells after 16-h treatment of the indicated ligands. **(E–G)** C1R cells (E) overexpressing HLA-A*02:01, (F) expressing wild-type level of MR1*01, or (G) overexpressing MR1.K43A mutant were incubated for the indicated periods with titrated quantities of ligand followed by flow cytometry. The C1R and C1R.A2 cells were stained with biotinylated 8F2F9 and W6/32, respectively, followed by PE-labeled streptavidin. Data in D–G are depicted as a fold change from basal surface expression upon incubation with the vehicle (1 mM NaOH). Each column represents the average of at least three independent experiments performed in duplicate with standard error (SEM) represented by error bars. One-way ANOVA statistical analysis was performed for all samples followed by Dunnett’s multiple comparison test using NaOH as a control (ns; not significant, ****P < 0.0001). Source data are available for this figure: SourceData FS1.

We then assessed the capacity of RF and RF catabolites to be loaded within recombinant MR1. The human MR1–β2-microglobulin (β2m) complex can only copurify when the MR1 Ag-binding pocket is occupied with ligand (Kjer-Nielsen et al., 2012). Photodegraded RF, as well as purified FMF, lumiflavin, lumichrome, and alloxazine, facilitated the correct folding of the MR1-β2m cocomplex in solution, while RF did not sponsor refolding of MR1 (Fig. S1, A and B). Next, we examined how these catabolites could stabilize MR1 using *in vitro* MR1-Ag complexes through a thermostability assay (Eckle et al., 2014) (Fig. 1 H). Here, the half-maximum melting temperatures (Tm50) of the stable complexes MR1-5-OP-RU and MR1-Ac-6-FP were 58.14 ± 0.60 and 64.22 ± 0.27°C, respectively, consistent with previous values (Eckle et al., 2014). MR1^K43A^-β2m, which refolds freely of Ag, cannot form a Schiff base, and is considered to be unstable, had a Tm50 of 45.65 ± 0.53°C. The RF catabolites had a moderate effect on stability of MR1 where MR1–photodegraded RF, MR1–lumiflavin, MR1–lumichrome, and MR1–alloxazine complexes had Tm50 values ranging from 49.89 to 52.02°C. Thus, the binding of these ligands stabilizes the MR1 protein but to a lower extent in comparison with MR1-5-OP-RU and MR1-Ac-6-FP (Fig. 1 H).

### RF-based catabolites downregulate MR1 cell surface expression

**Figure 2.**
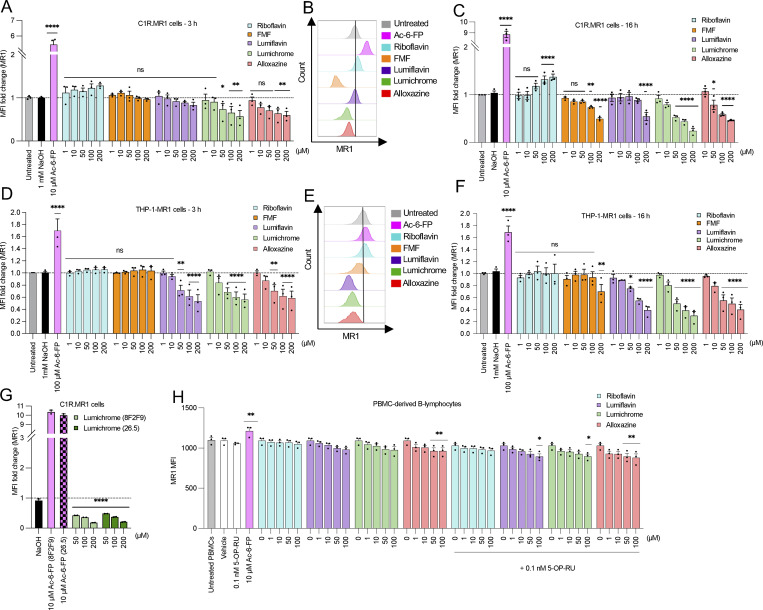
**RF catabolites modulate MR1 expression on the surface of APCs. (A–C)** Bar graphs depict the expression of surface MR1*01 on C1R.MR1 cells incubated for 3 h (A) or 16 h (B and C) with titrated quantities of ligands followed by staining with 8F2F9 anti-MR1 antibody. Shown in B are overlay histograms for the expression of surface MR1*01 on C1R.MR1 cells after 16-h treatment with 200 µM of RF catabolites. **(D–F)** MR1 was then quantified after adding the indicated concentrations of the RF catabolites on THP-1.MR1 cells incubated for 3 h (D) or 16 h (E and F). Shown in E are overlay histograms for the expression of surface MR1*01 on THP-1.MR1 cells after 16-h treatment with 200 µM of RF catabolites. **(G)** Bars indicate the expression of surface MR1 on C1R.MR1 cells after treatment with lumichrome overnight and then staining with 26.5 vs. 8F2F9 MAb for comparison. **(H)** MR1 expression on the surface of PBMC-derived B lymphocytes was measured after treatment with RF catabolites for 16 h in the presence or absence of 5-OP-RU. Data in A–G represent the gMFI fold change from three independent experiments performed in duplicates, with standard error (SEM) represented by the error bars. Data in H are the average of three independent experiments done on PBMCs from three different donors performed in duplicate, with standard error (SEM) represented by the error bars. One-way ANOVA statistical analysis was performed for all samples with Dunnett’s multiple comparisons performed using NaOH as a control (ns: not significant, *P < 0.05, **P < 0.01, ****P < 0.0001). Statistical analysis for the 5-OP-RU competition experiment (H) used 5-OP-RU as a control. MR1, MHC-I–related protein. gMFI, geometric mean fluorescence intensity.

Next, we investigated whether these RF catabolites can impact MR1 cell surface expression in both MR1-transduced B cell lymphoblastoid (C1R.MR1) and monocytic (THP-1.MR1) cell lines (Fig. 2). Here, the cells were incubated with 1–200 µM of RF or the RF catabolites, and the cell surface levels of MR1 were quantified by flow cytometry (Awad et al., 2020a; Keller et al., 2017). Cell viability was unaffected, even at the highest concentrations of the catabolites, as depicted in Fig. S1 D. In C1R.MR1 cells, RF—up to 200 µM—did not alter MR1 expression from the baseline after 3 h (Fig. 2 A) but modestly upregulated MR1 after 16 h (Fig. 2, B and C), whereas none of the RF catabolites could upregulate MR1 cell surface expression at either time point. Instead, lumichrome and alloxazine reduced MR1 cell surface after 3 h (Fig. 2 A), while all RF catabolites downregulated MR1 after 16 h in a concentration-dependent manner (Fig. 2, B and C). The MR1 downregulation seen in C1R.MR1 cells was specific to MR1 as the level of HLA-A*02:01 was not affected after incubating C1R-A2 with the ligands (Fig. S1 E). A similar trend was observed after incubating THP-1-MR1 with RF catabolites for 3 and 16 h (Fig. 2, D–F), yet lumiflavin downregulated MR1 after 3 h (Fig. 2 D), in comparison with C1R.MR1 cells. This MR1 downregulation varied between cell types, with a higher extent observed in THP-1.MR1 than C1R.MR1, and was seen after staining with two different conformational anti-MR1 antibodies (clones 8F2F9 and 26.5; Fig. 2 G). Beyond APCs overexpressing MR1, we also measured MR1 on the surface of cells expressing wild-type levels of MR1. While no impact was observed after 3 h when the parental C1R cells were incubated with these RF catabolites (Fig. S1 F), MR1 expression on primary human B cells was altered as was observed for C1R.MR1 and THP-1.MR1 cells. Here, B cells were treated with varied doses of RF catabolites and the highest doses of lumiflavin, lumichrome, and alloxazine reduced MR1 surface expression, particularly when coincubated with 5-OP-RU (Fig. 2 H). Overall, our data suggest that the direct interactions of RF catabolites with MR1 in the cells lead to the reduction of cell surface expression where lumichrome is the most potent downregulator followed by alloxazine, lumiflavin, and then FMF.

### MR1-binding RF catabolites keep MR1 in the ER in an immature state

**Figure 3.**
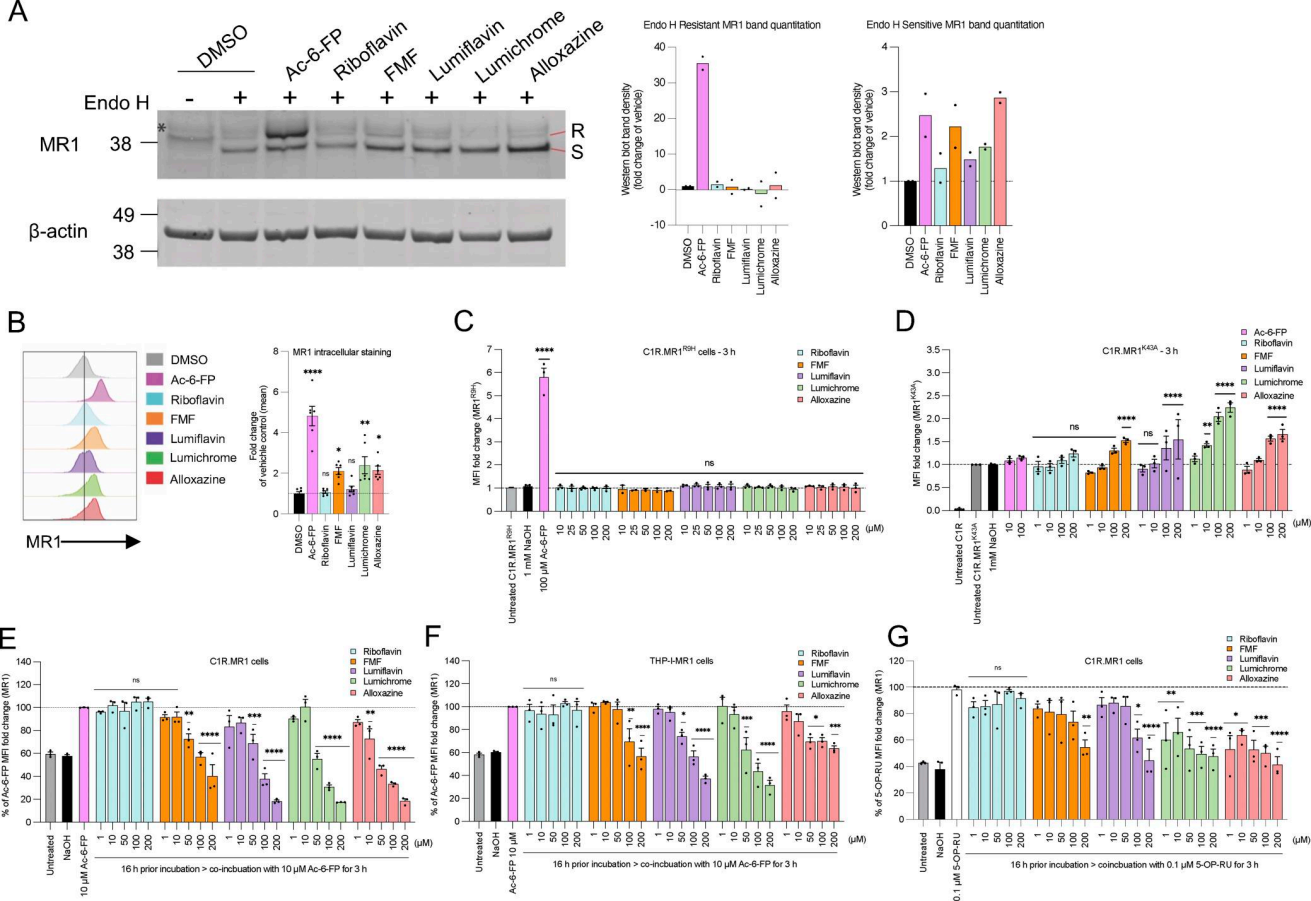
RF catabolites induce retention of MR1*01 in the ER, but not MR1 ^**K43A**^ **. (A)** Analysis of Endo H–treated (+) or untreated (−) MR1 by western blotting with anti-MR1 (8G3) after culturing C1R.MR1 cells with DMSO (vehicle control), Ac-6-FP (10 μM), RF (100 μM), FMF (100 μM), lumiflavin (100 μM), lumichrome (100 μM), or alloxazine (100 μM) for 16 h, at 37°C. S, Endo H–susceptible MR1; R, Endo H–resistant MR1. The bars show the Endo H–susceptible MR1 and Endo H–resistant MR1 fractions quantified in at least two independent experiments. **(B)** Intracellular total MR1 level was also measured in C1R.MR1 cells by flow cytometry after treating the cells with the indicated ligands (100 µM) for 16 h followed by permeabilization and staining with anti-MR1-PE (8F2.F9). Shown are the overlay histograms (left) and a bar chart depicting the gMFI fold change of intracellular MR1 level (right) from three independent experiments performed in duplicates, with standard error (SEM) represented by the error bars. **(C and D)** C1R cells expressing (C) MR1^R9H^ mutant or (D) MR1^K43A^ mutant were incubated for the indicated periods with titrated quantities of ligand followed by flow cytometry. **(E–G)** Competition between RF catabolites and Ac-6-FP in E C1R.MR1 cells and (F) THP-I.MR1 cells, as well as (G) 5-OP-RU in C1R.MR1 cells, was quantified after incubation with the indicated concentrations of RF catabolites for 16 h before the addition of Ac-6-FP/5-OP-RU for further 3 h. Shown in E–G is the average percentage reduction in Ac-6-FP/5-OP-RU–induced MR1 upregulation in three independent experiments performed in duplicates with standard error (SEM) represented by error bars. One-way ANOVA statistical analysis was performed for all samples with Dunnett’s multiple comparisons performed using NaOH, Ac-6-FP, or 5-OP-RU as controls for the comparison (ns: not significant, *P < 0.05, **P < 0.01, ***P < 0.001, ****P < 0.0001). gMFI, geometric mean fluorescence intensity. Source data are available for this figure: SourceData F3.

The downregulation of MR1 on the cell surface by the RF catabolites could be due to the depletion of the intracellular ER-resident MR1 pool, or due to the retention of MR1 in the ER, as shown for the DB28 ligand (Salio et al., 2020). To investigate this, C1R.MR1 cells were treated for 16 h with 100 µM RF or RF catabolites, conditions that induce MR1 downregulation (Fig. 3 A). Cells were lysed, and treated with endoglycosidase (Endo) H, to distinguish molecules that remain within the ER from those that have trafficked to the cell surface (McWilliam et al., 2016), and then, MR1 levels were assessed by immunoblotting (Fig. 3 A). In the absence of exogenous ligands, the majority of MR1 remains predominantly Endo H–sensitive, as demonstrated by the dominant band of lower molecular weight on SDS-PAGE. The addition of Ac-6-FP, which triggers MR1 egression from the ER, results in an increase in MR1 of a higher molecular weight, in line with Endo H resistance (Fig. 3 A). Neither RF nor its catabolites induced any Endo H–resistant MR1; however, RF catabolites consistently increased the level of Endo H–sensitive MR1 molecules. This indicates that these RF catabolites do not deplete ER-resident MR1 but rather retain and induce its accumulation.

Previously, it was shown that within the ER, there are two forms of MR1—“folded” and “open”—and these can be distinguished with a conformationally sensitive antibody 8F2.F9, which specifically recognizes the folded form (McWilliam et al., 2016; McWilliam et al., 2020). The open MR1 is not bound to β2m, whereas the folded MR1 is associated with β2m, is more stable, and shows less degradation in the absence of ligands (McWilliam et al., 2016; McWilliam et al., 2020). To determine whether the RF catabolites increase the level of either form of ER-resident MR1 molecules, C1R.MR1 cells were treated with or without RF and its catabolites as above, and the levels of folded MR1 were detected by intracellular staining with 8F2.F9 and detected by flow cytometry (Fig. 3 B). As expected, Ac-6-FP significantly increased total folded MR1, which includes both intracellular and cell surface complexes. The RF catabolites FMF, lumichrome, and alloxazine, but not RF or lumiflavin, also increased the total level of folded MR1 (Fig. 3 B).

This suggests that FMF, lumichrome, and alloxazine induce the folded conformation of intracellular MR1 molecules. This is consistent with the hypothesis that these metabolites bind to open ER-resident MR1 molecules and induce their folding to the more stable “closed” conformation. This stabilizes these complexes without inducing their release from this compartment, leading to their intracellular accumulation. This provides a mechanistic explanation for the observed downregulation of MR1 from the cell surface in the presence of these metabolites.

### Requirements for RF catabolite–mediated down-modulation of MR1 cell surface levels

We then tested the effect of the RF catabolites on the expression of two mutant versions of MR1 termed MR1^R9H^ (Howson et al., 2020) and MR1^K43A^ (Reantragoon et al., 2013). First, we tested the MR1^R9H^ mutant (Howson et al., 2020), which represents a rare, natural human polymorph. It lacks the ability to bind and present RF metabolites such as 5-OP-RU yet retains the ability to bind other ligands such as folate metabolite Ac-6-FP, offering an opportunity to further understand the molecular constraints on MR1 binding to RF derivatives. We used C1R.MR1^R9H^ cells, which are produced by stable transduction of wild-type C1R cells, which express low levels of wild-type MR1, with a vector expressing the MR1^R9H^ mutant (Howson et al., 2020). Although 8F2F9 anti-MR1 mAb does not distinguish between WT MR1 and MR1^R9H^, the majority of MR1 on the cell surface in this context is MR1^R9H^. As expected, the level of MR1^R9H^ on the surface of C1R.MR1^R9H^ significantly increased after Ac-6-FP treatment (Fig. 3 C). When C1R. MR1^R9H^ cells were incubated with RF and RF catabolites, the MR1^R9H^ surface level did not deviate from the background level (Fig. 3 C). In addition, lumichrome did not refold with MR1^R9H^ in solution. Thus, similar to microbial 5-OP-RU, RF catabolites also do not bind MR1^R9H^. Next, we sought to understand how the previously characterized K43A mutation affects ligand binding. This mutation stabilizes MR1 and helps refolding in the absence of exogenous ligands (Reantragoon et al., 2013), and thus, the level of trafficked MR1^K43A^ is significantly increased on the surface of C1R.MR1^K43A^ cells (Fig. 3 D). Surprisingly, lumichrome, alloxazine, and lumiflavin increased MR1^K43A^ at the cell surface after 3 h (Fig. 3 D) and this MR1 upregulation continues with lumichrome and RF after 16 h (Fig. S1 G). These data are in alignment with our data showing MR1 binding and ER retention, whereby the RF catabolites bind MR1 and in the wild-type setting induce MR1 retention, which results in reduced surface expression; however, in the case of the K43A mutation, MR1 retention is disrupted, as previously described (Reantragoon et al., 2013), and thus, these MR1-binding ligands result in greater ER egress and higher surface expression in this artificial setting.

### MR1-binding RF catabolites compete with VitBAgs for MR1 binding

We next tested whether FMF, lumiflavin, lumichrome, and alloxazine impact the MR1 cell surface expression induced by Ac-6-FP or 5-OP-RU. The RF catabolites were incubated with C1R.MR1 and THP-1.MR1 cells overnight at 1–200 µM concentration before the addition of either 10 µM Ac-6-FP or 5-OP-RU, then coincubated for another 3 h. RF itself did not modulate Ac-6-FP or 5-OP-RU responses (Fig. 3, E–G). In comparison, the four RF catabolites reduced the Ac-6-FP–induced cell surface upregulation of MR1 in a concentration-dependent manner, more profoundly in C1R.MR1 (Fig. 3 E) than THP-1.MR1 cells (Fig. 3 F). Interestingly, the highest concentration (200 µM) of lumiflavin, lumichrome, and alloxazine could reduce more than 80% of the Ac-6-FP response. The competition with 5-OP-RU was less potent with tested concentrations of the RF catabolites in C1R.MR1 cells (Fig. 3 G), in line with the stronger affinity of 5-OP-RU for MR1, compared with Ac-6-FP, as measured by the fluorescence polarization assay (Fig. 1 B). These cellular data, along with the moderate affinity for MR1 revealed by the fluorescence polarization assay, suggest that FMF, lumiflavin, lumichrome, and alloxazine are able to bind MR1 molecules within cells and hinder the capacity of other potent ligands to stimulate MR1 trafficking to the cell surface.

### MR1-binding RF catabolites selectively inhibit MAIT cell effector functions

**Figure S2.**
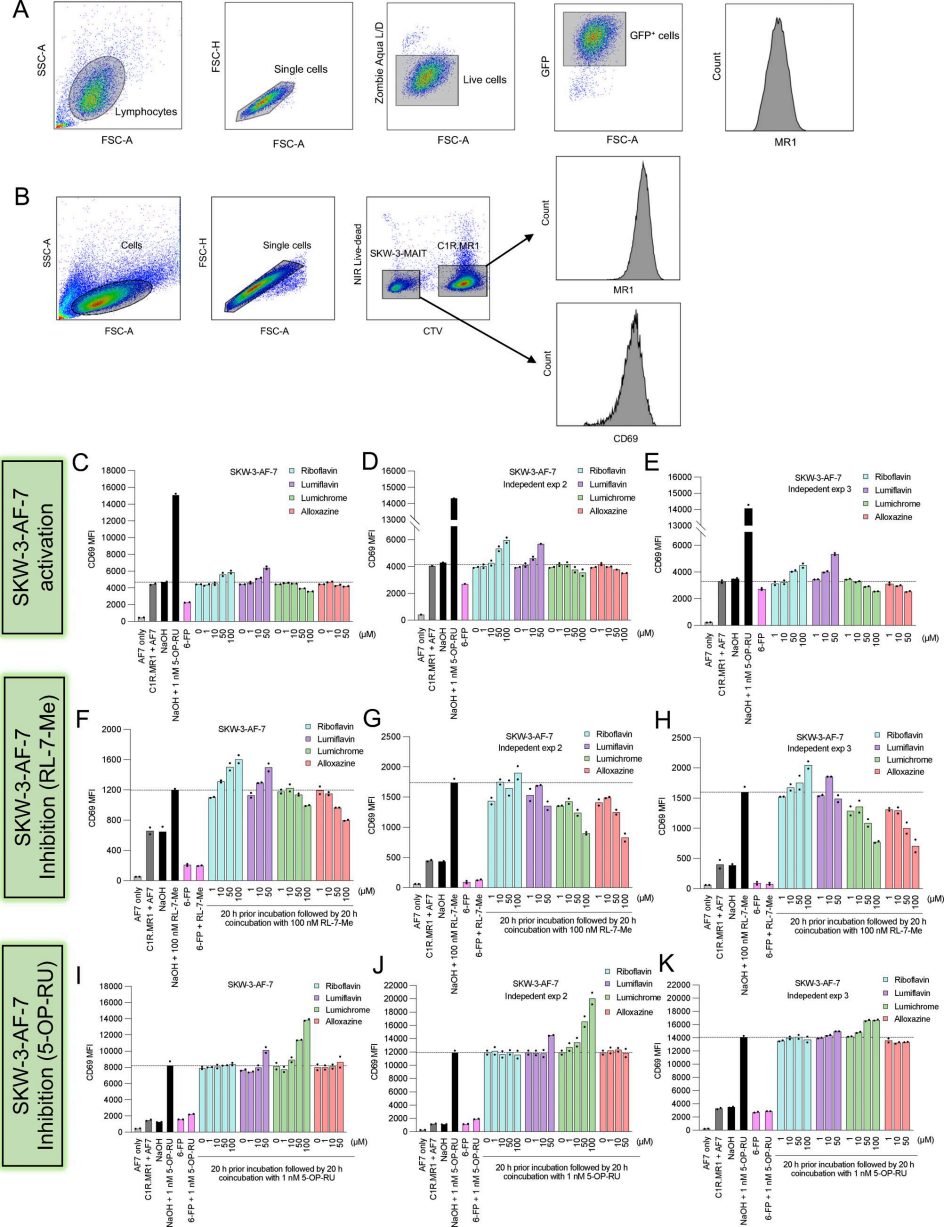
**Gating strategies of flow cytometry cell line experiments. (A and B)** Shown are the gating strategies used in (A) MR1 upregulation experiments and (B) T cell activation experiments. **(C–K)** Bar graphs show three independent biological replicates for the CD69 expression measured on the surface of SKW-3 overexpressing the typical MAIT cell clones AF-7 (TRAV1-2^+^ TRBV6-1^+^) cocultured with C1R.MR1 cells in the absence (C–E) or presence of RL-7-Me (F–H) or 5-OP-RU (I–K) after preincubation with titrated quantities of indicated ligands. Individual data points in C–K are technical replicates within the same experiment.

**Figure S3.**
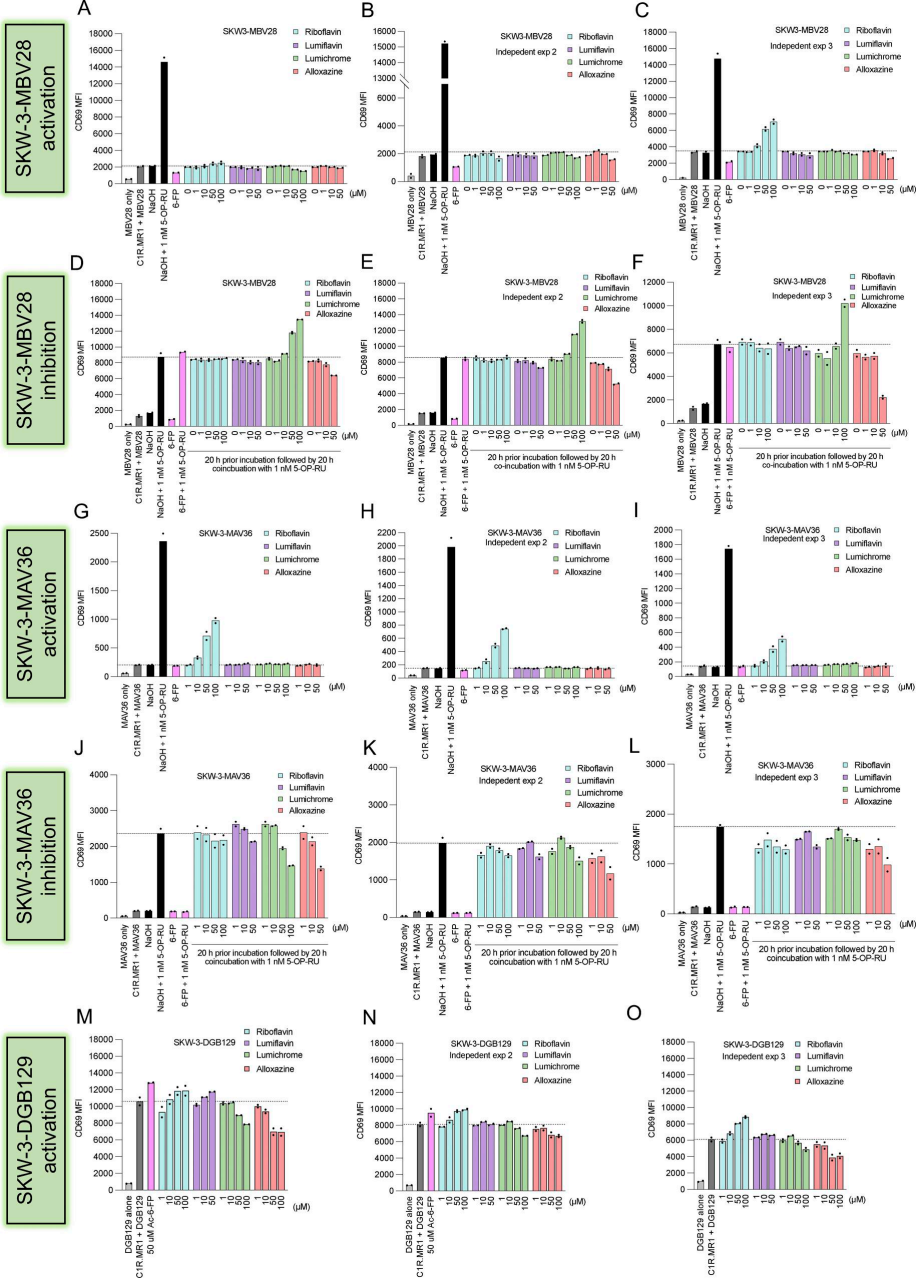
**RF-based catabolites impact on *in vitro* MR1-dependent T cell activation. (A–O)** Bar graphs show three independent biological replicates for the CD69 expression measured on the surface of SKW-3 overexpressing the typical MAIT cell clone (A–F) MBV28 (TRAV1-2^+^ TRBV28^+^), (G–L) the atypical MAIT MAV36 (TRAV36^+^ TRBV28^+^), or (M–O) the MR1 autoreactive clone DGB129 (TRAV29^+^ TRBV12-4^+^) cocultured with C1R.MR1 cells in the absence (labeled “activation”) or presence of (labeled “inhibition”) 5-OP-RU after preincubation with titrated quantities of indicated ligands. Individual data points are technical replicates within the same experiment.

Given the intracellular MR1 retention induced by the RF catabolites, we anticipated that these ligands could influence the activity of MR1-reactive T cells. First, we utilized a system whereby reporter SKW-3 T cell lines were cocultured with C1R.MR1 APCs and activation was determined by CD69 expression. While SKW-3 cells expressing two distinct MAIT TCR clones (AF-7 and MBV28) were not activated by RF or its catabolites (Fig. S2, C–E and Fig. S3, A–C), these ligands differentially modulated the responses of these cells to two microbial RF metabolite Ags, RL-7-Me and 5-OP-RU (Fig. S2, F–K and Fig. S3, D–F). For example, while SKW-3-AF-7 reactivity to RL-7-Me was inhibited in a dose-dependent manner by lumichrome and alloxazine (Fig. S2, F–H), activation was enhanced in response to RF and lumiflavin. Similarly, 5-OP-RU reactivity was not altered by RF or alloxazine, whereas lumiflavin and lumichrome enhanced activation (Fig. S2, I–K). In contrast, SKW-3-MBV28 cells were inhibited by alloxazine but activated by lumichrome (Fig. S3, D–F). Two MR1-restricted TRAV1-2^−ve^ SKW-3 T cell lines were also tested. Here, both the nucleobase-adduct reactive SKW-3-DGB129 line and reactivity to 5-OP-RU by the SKW-3-MAV36 line were inhibited by lumichrome and alloxazine (Fig. S3, G–O).

**Figure 4.**
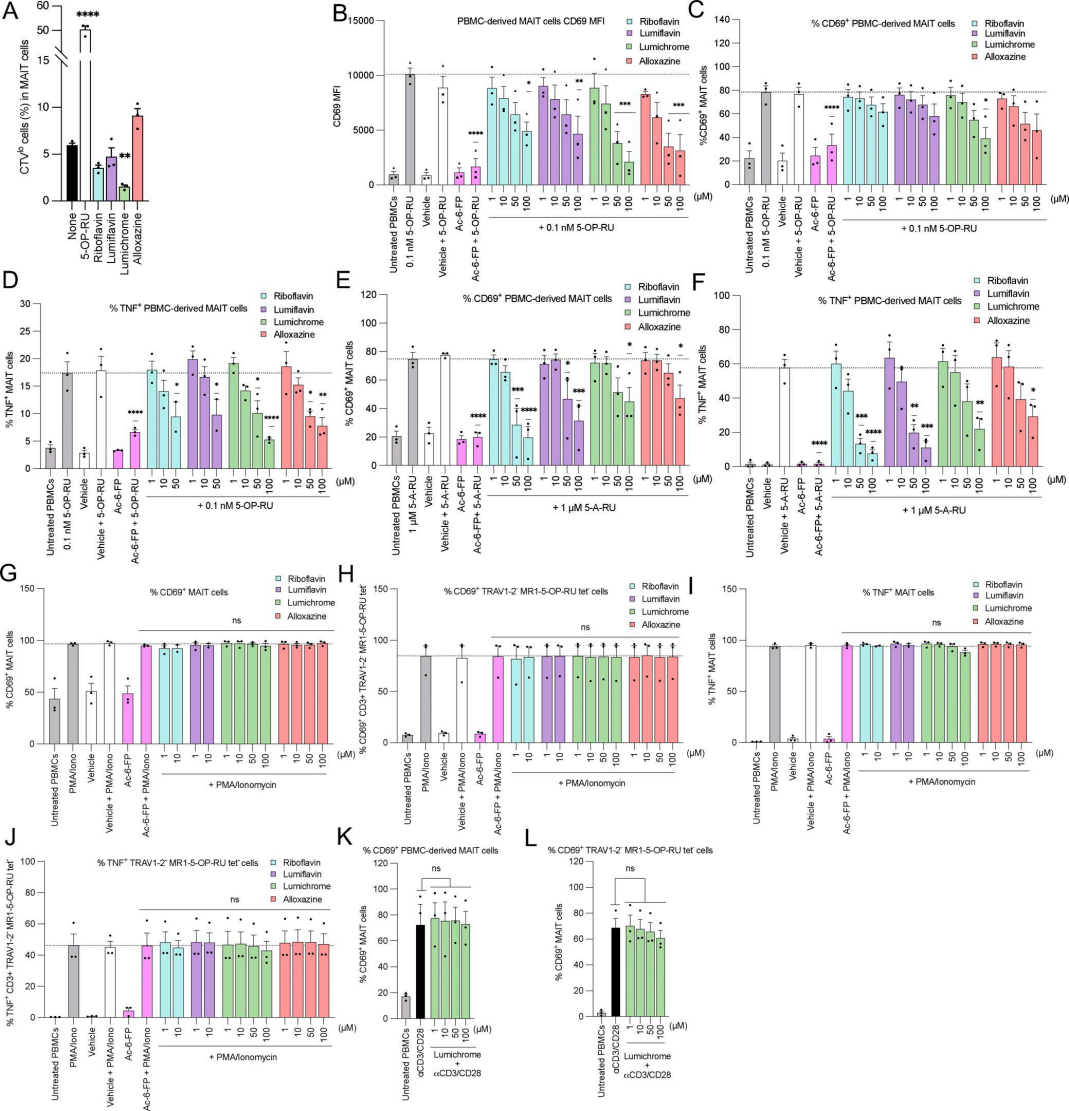
**RF catabolites selectively inhibit *ex vivo* human MAIT cell activity. (A)** Bar graph showing the proportion of CTV^low^ MR1-5-OP-RU tetramer^+^ MAIT cells as a proportion of total MAIT cells in PBMC after 7-day culture in the presence of 5-OP-RU (10 µM), RF (100 µM), lumiflavin (100 µM), lumichrome (100 µM), or alloxazine (100 µM) on day 7. **(B–D)** Bar graphs showing (B) MFI CD69, (C) proportion of CD69^+^, or (D) proportion of TNF^+^ MAIT cells from PBMCs coincubated with titrated doses of RF or RF catabolites in the presence of 5-OP-RU. **(E and F)** Bar graphs showing (E) proportion of CD69^+^ or (F) proportion of TNF^+^ MAIT cells from PBMCs coincubated with titrated doses of RF or RF catabolites in the presence of 5-A-RU. **(G–J)** Bar graphs showing (G) the proportion of CD69^+^ MAIT cells, (H) the proportion of CD69^+^ TRAV1-2^−ve^ MR1-5-OP-RU tetramer^−ve^ cells, (I) the proportion of TNF^+^ MAIT cells, or (J) the proportion of TNF^+^ TRAV1-2^−ve^ MR1-5-OP-RU tetramer^−ve^ cells from PBMCs preincubated with titrated doses of RF or RF catabolites followed by stimulation with PMA and ionomycin. **(K and L)** Bar graphs showing the proportion of CD69^+^ (K) MAIT cells or (L) TRAV1-2^−ve^ MR1-5-OP-RU tetramer^−ve^ cells, from PBMCs preincubated with titrated doses of RF or RF catabolites followed by stimulation with αCD3/αCD28-coated beads. Data from all graphs represent the average of three independent experiments performed in duplicate on PBMCs from three different donors. The error bars represent the standard error of the mean (SEM). One-way ANOVA statistical analysis was performed for all samples with Dunnett’s multiple comparisons performed using vehicle, 5-OP-RU, or 5-A-RU as controls (ns: not significant, *P < 0.05, **P < 0.01, ***P < 0.001, ****P < 0.0001).

**Figure S4.**
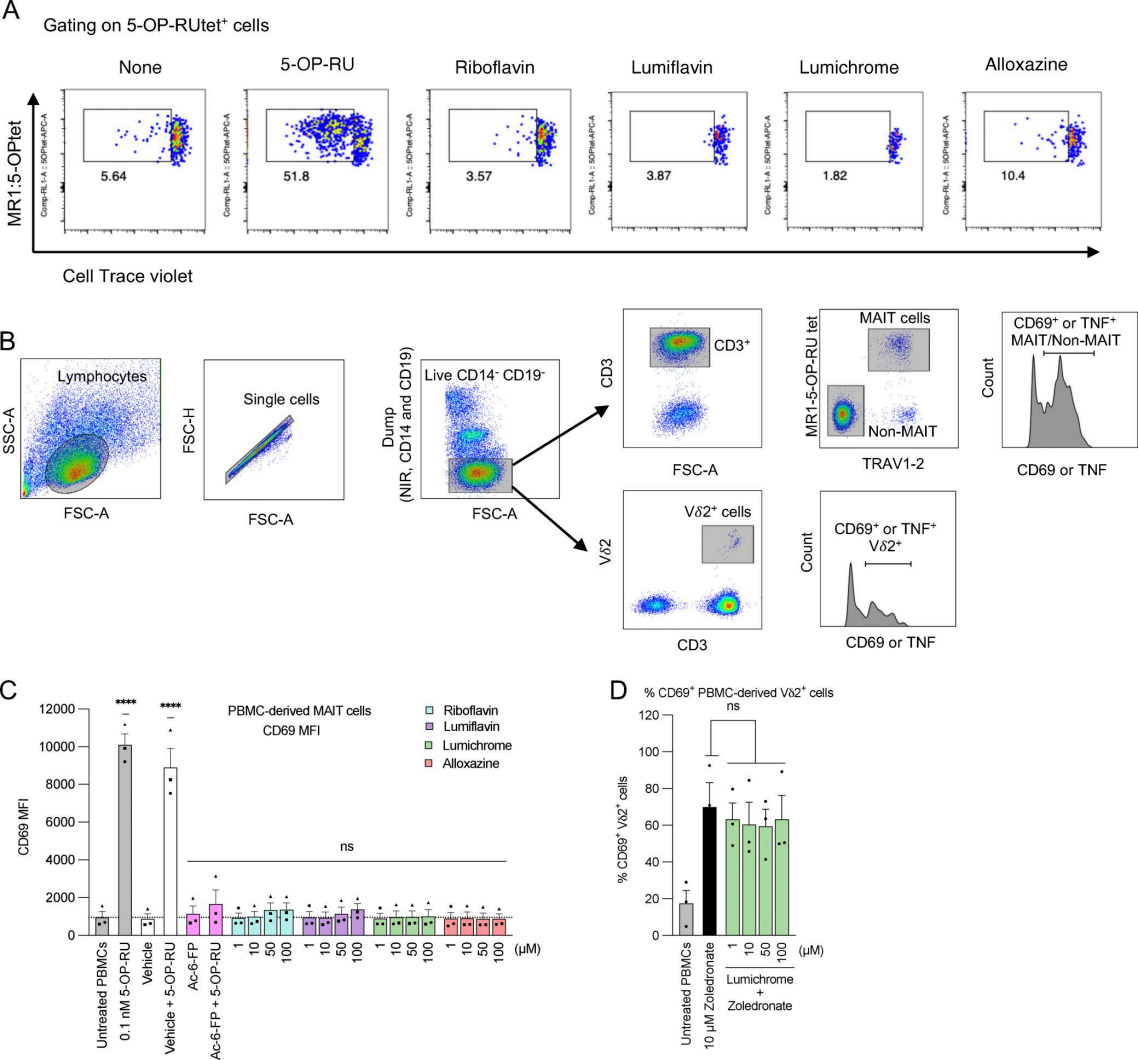
**Gating strategy and activation of PBMCs. (A)** Representative flow cytometry dot plots of proliferating CD3^+^MR1−5-OP-RU tet^+^CTV^lo^ MAIT cells related to Fig. 4 A. **(B)** Shown in B is the gating strategy used in PBMC experiments. **(C)** Human PBMCs were titrated with indicated ligands for 16 h, and then, CD69 MFI was measured on the surface of gated MAIT cells. **(D)** PBMCs were titrated with lumichrome in the presence of zoledronate, and then, % CD69^+^ Vδ2^+^ cell population was measured. Data in C and D represent the average of three independent experiments performed in duplicate on PBMCs from three different donors. The error bars represent the standard error of the mean (SEM). One-way ANOVA statistical analysis was performed for all samples using Dunnett’s multiple comparisons (ns: not significant, ****P < 0.0001).

While these results provide some evidence of inhibition of MR1-mediated Ag recognition by these catabolites to both high- and low-potency ligands (5-OP-RU and RL-7-Me, respectively [Awad et al., 2020a]), in some instances there was unexpected induction of activation. We reasoned that these unexpected results are likely a result of the confounding high over-expression of MR1, enabling surface exchange of these ligands, which resulted in both the bypassing of MR1 ER retention and allowing different TCRs to respond to surface-loaded MR1 in a clone-dependent manner. Therefore, we next employed an assay system that uses primary APCs and MAIT cells, which more closely mimic physiological Ag presentation conditions. Here, we cocultured healthy human peripheral blood mononuclear cells (PBMCs) with RF or RF catabolites and assessed the effects on MAIT cells. First, MAIT proliferation was measured after CellTrace Violet (CTV)–labeled PBMCs were cocultured with various RF ligands. CD3^+^ MR1-5-OP-RU tetramer^+^ (tet^+^) MAIT cells significantly proliferated upon stimulation with 5-OP-RU. Lumichrome significantly reduced MAIT cell proliferation in comparison with the other RF catabolites, which did not significantly change the baseline level (Fig. 4 A and Fig. S4 A). MAIT activation was then evaluated by quantifying CD69 expression on the surface of gated MAIT cells after 16-h incubation with RF and its catabolites. The gating strategy we used is shown in Fig. S4 B. While 5-OP-RU induced robust CD69 upregulation, none of the RF catabolites induced CD69 upregulation beyond that of the known MAIT antagonist Ac-6-FP (Fig. S4 C).

**Figure 5.**
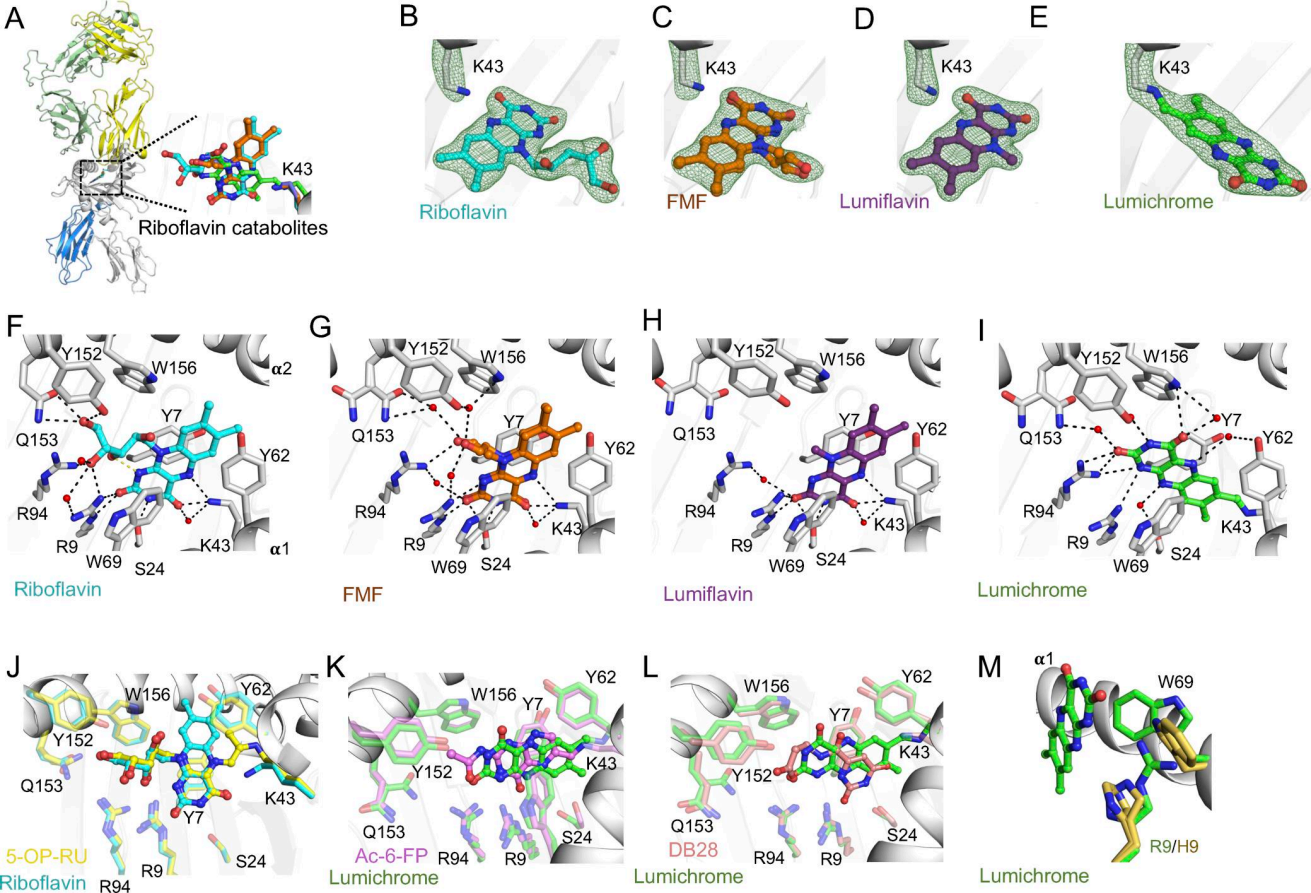
**Overall docking and molecular interactions of RF and RF catabolites within MR1 A′-pocket. (A)** Superposition of the TCR-MR1-RF catabolite crystal structures showing the RF and its catabolites within the MR1-binding cleft of the MR1-RF structure interacting with MR1-Lys43. **(B–E)** Electron density omit maps (green mesh) of (B) RF, (C) FMF, (D) lumiflavin, and (E) lumichrome contoured at 2σ. **(F–J)** Molecular contacts of (F) RF, (G) FMF, (H) lumiflavin, and (I) lumichrome with the residues of MR1-A′-pocket in the MR1-Ag structures. Shown in J is the superposition of RF (cyan) with 5-OP-RU (yellow; PDB: 6PUC), which both have ribityl tail that extends toward the α2 helix. **(K–L)** Superposition of lumichrome (green) with (K) Ac-6-FP (pink; PDB: 4PJ5) and (L) DB28 (salmon; PDB: 6PVC) within MR1 ligand-binding cleft. **(M)** Superposition of lumichrome within the MR1 ligand-binding pocket of MR1^R9H^ structure (PDB: 6W9V) showing the MR1 residues from MR1–lumichrome structure in green and MR1^R9H^ residues in yellow. MR1 and β2m are colored white and marine, respectively. Here, the cutoff for hydrogen bonds, salt bridges, and VDW interactions was set at 3.5 Å, 4 Å, and 4 Å, respectively. Ligands are colored as follows: RF, cyan; FMF, orange; lumiflavin, magenta; and lumichrome, green. The α and β chains of the AF-7 TCR are colored yellow and pale green, respectively.

Next, PBMCs were incubated for 18 h with the MAIT agonists 5-OP-RU (Fig. 4, B–D) or 5-A-RU (Fig. 4, E and F), in the presence of varied concentrations of RF and RF catabolites. Unlike the SKW-3-MAIT–based system, we observed concentration-dependent competition between RF catabolites and the MAIT agonists leading to consequent MAIT inhibition. This was reflected by the reduction of the mean fluorescence intensity (MFI) of CD69 on activated MAIT cells (Fig. 4 B), the percentage of CD69^+^ MAIT cells (Fig. 4, C and E), and MAIT cell TNF production (Fig. 4, D and F), where lumichrome was the most potent competitor showing the strongest MAIT cell inhibition. This blockade was MAIT TCR-dependent, as neither RF nor RF catabolites could block CD69 upregulation nor TNF production by MAIT or non-MAIT TRAV1-2^−ve^ cells in response to TCR-independent stimuli PMA and ionomycin (Fig. 4, G–J). Similarly, CD69 upregulation by MAIT or TRAV1-2^−ve^ cells was not reduced in response to αCD3- and αCD28-coated beads by lumichrome as a model RF catabolite (Fig. 4, K and L). Furthermore, lumichrome did not interfere with the Vγ9Vδ2 T cell activation induced by zoledronate, which activates these cells in an MR1-independent manner, using the same *in vitro* culture assay (Fig. S4 D). Collectively, these data show that RF catabolites, especially lumichrome, can bind to MR1, retain MR1 in the ER, and thus act as specific TCR-mediated antagonists for MAIT cells.

### Crystal structures of MR1-RF–derived ligands

To understand the structural basis for the binding of RF and RF catabolites to MR1, we determined the high-resolution crystal structures of MR1 in complex with RF, photodegraded RF, FMF, lumiflavin, and lumichrome (Fig. 5 and Table S1). Crystallization of MR1 binary structures is challenging, so we used the MAIT A-F7 TCR (Tilloy et al., 1999) to aid crystallization, as reported previously (McInerney et al., 2024; Salio et al., 2020). In the MR1–photodegraded RF, the electron density within the MR1 A′-pocket is large suggesting the presence of more than one ligand (Fig. S1 C), in line with the mass spectrometry data, so we did not refine this further. The AF-7 TCR acquired the typical docking mode previously seen in MR1–ligand–AF-7 ternary structures (Fig. 5 A). The electron density for the remaining ligands within the MR1 A′-pocket was unambiguous (Fig. 5, B–E), thereby permitting detailed structural analysis. In accommodating these three-ringed ligands (Fig. 5, F–I), minimal conformational changes within the pocket residues were observed, and the side chains of the MR1 Ag-binding residues were mostly conserved. Notably, lumichrome was the only ligand that formed a covalent interaction with MR1-Lys43 (Fig. 5, B–E). The volume of the MR1 A′-pocket in the crystal structures of MR1-RF, MR1-FMF, MR1–lumiflavin, and MR1–lumichrome was 960, 1020, 970, and 970 Å^3^, respectively (calculated using the CASTpFold online tool), suggesting that no significant changes were observed in the overall structure of the MR1 protein itself.

In the crystal structure of MR1-RF, RF did not form a Schiff base with MR1-Lys43 (Fig. 5 B); yet the 4-carbonyl and 5-nitrogen in the flavin (isoalloxazine) ring formed H-bonds with Lys43 (Fig. 5 F). The 2-carbonyl and 3-nitrogen groups of the RF ring formed H-bonds with MR1-Arg9 and Ser24, respectively. The ribityl moiety formed H-bonds with MR1-Arg9, Arg94, Tyr152, and Gln153, in addition to van der Waals (VDW) interaction with MR1-Trp156 (Fig. 5 F). The positioning of the ribityl tail is governed by a network of intramolecular and intermolecular polar contacts. The intramolecular interaction is formed between the 3′-OH group and the isoalloxazine ring (Fig. 5 F). In addition, Tyr152 and Gln153 of MR1 formed hydrogen bonds with the 5′-OH group. RF adopted a very similar orientation to the potent MAIT activator 5-OP-RU in the MR1 pocket (Fig. 5 J), leading to the same positioning of the residues in the cleft except for a slight shift in Tyr62 orientation toward the periphery of the pocket to accommodate RF.

In the crystal structures of MR1-FMF and MR1-lumiflavin, the flavin rings of both FMF and lumiflavin ligands had the same orientation as for RF and as such were involved in a similar network of interactions (Fig. 5, G and H). The acetyl moiety in FMF adopted two conformations in the pocket. This acetyl moiety allowed the formation of an H-bond with MR1-Arg94, as well as water-mediated interactions with the α2 residues Tyr152, Gln153, and Trp156 (Fig. 5 G). Here, the lack of ribityl tail in both FMF and lumiflavin disfavored the formation of “direct” hydrogen bonding with MR1-α2 residues (Fig. 5, G and H). This shows clearly that RF catabolites are well positioned within the MR1 A′-pocket, allowing the stabilization of MR1 in the ER and reduced trafficking to the cell surface.

### Lumichrome forms a flavin bond with MR1-Lys43

**Figure S5.**
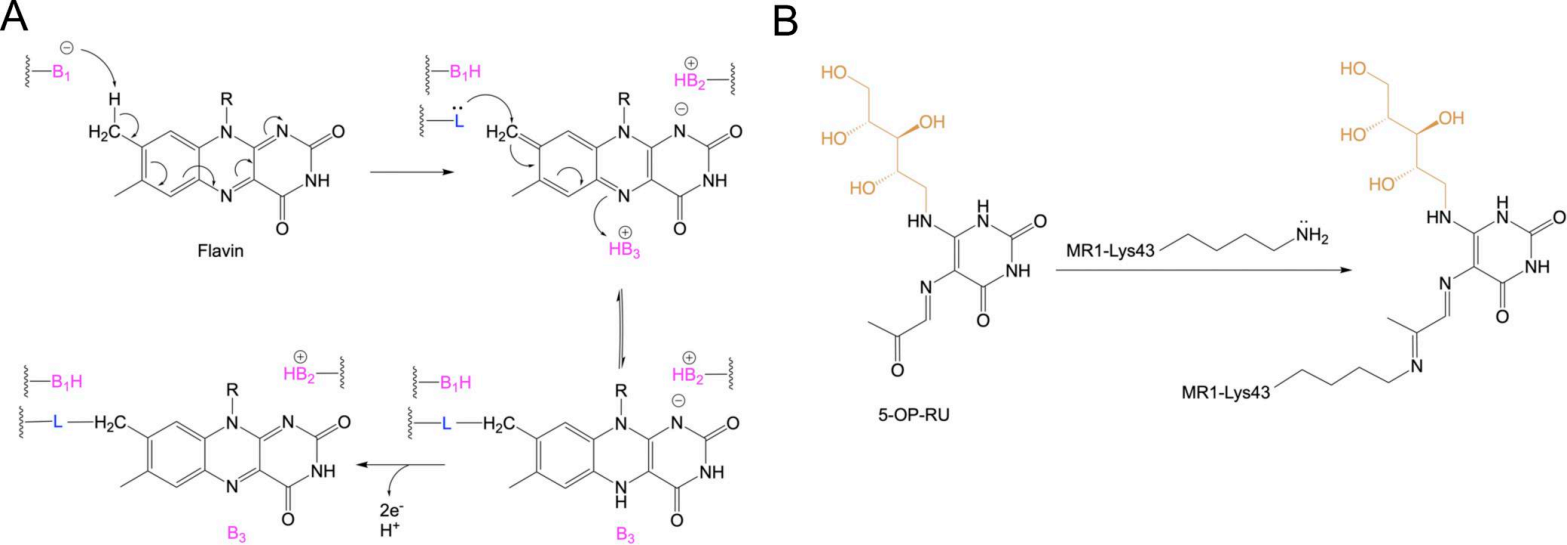
**Comparison between the flavin bond and Schiff base formation. (A)** Shown is the proposed general mechanism for the formation of the covalent flavin–protein bond at the isoalloxazine ring (adapted from Heuts et al. [2009]). Here, B_1_, B_2_, and B_3_ are basic side chains, while L^^−^^ is the nucleophilic side chains. **(B)** Schiff base is formed through imine condensation between the carbonyl group of a ligand (here, 5-OP-RU) and the amino group of MR1-Lys43.

Lumichrome was sequestered within the MR1 aromatic cradle where its isoalloxazine ring sat in a perpendicular orientation to that of the other RF catabolites (Fig. 5 I). This lumichrome ring orientation is similar to the potent MR1 upregulators Ac-6-FP and 6-FP (Eckle et al., 2014) (Fig. 5 K), ruling out that the downregulation observed with lumichrome is due to changes in lumichrome docking inside MR1. Here, the flavin ring of the lumichrome ligand was sandwiched between MR1-Tyr7, Tyr62, Trp69, and Trp156, while the 2-carbonyl moiety formed H-bonds with MR1-Arg94, water-mediated H-bonds with MR1-Gln153, and VDW interactions with MR1-Arg9 (Fig. 5 I). Also, the 4-carbonyl moiety formed a direct H-bond with MR1-Trp156. The amino groups of the flavin ring formed direct H-bonds with MR1-Arg9 and Arg94, as well as water-mediated H-bonds with MR1-Ser24, Tyr62, and Try152. Surprisingly, a unique MR1–Lys43–flavin ring linkage flavin bond was observed between the 7-methyl group of the lumichrome ring and MR1-Lys43 (Fig. 5 I). To compare, the MR1 ligand DB28 could not form a covalent interaction with MR1-Lys43 (Salio et al., 2020), though the orientations of MR1 pocket residues are comparable to that of the lumichrome structure (Fig. 5 L) explaining a similar downregulation trend. As lumichrome was unable to refold with the MR1^R9H^ mutant, MR1–Arg9–lumichrome interaction is most likely essential for the ligand stabilization (Fig. 5 M) and MR1 downregulation (Fig. 3 C). The mechanisms of covalent flavination and the possible roles of covalent protein–flavin bonds have been previously reported in a significant number of flavoenzymes, which use a covalently protein-bound flavin cofactor (Heuts et al., 2009; Wang et al., 2022b). A proposed mechanism for the covalent binding of lumichrome to MR1 is depicted in Fig. S5.

## Discussion

Ligands derived from by-products of microbial RF (vitamin B_2_; RF) biosynthesis are the most well-characterized MR1 binders and highly potent MAIT cell stimulators (Awad et al., 2023; Corbett et al., 2014; Kjer-Nielsen et al., 2012). These Ags are derived from the microbe-specific RF intermediate, 5-A-RU (Corbett et al., 2014). RF itself, however, is also essential for humans and is typically derived from dietary sources. Here, RF is necessary for the synthesis of the flavin coenzymes, including flavin mononucleotide (FMN) and flavin adenine dinucleotide (FAD), which have key roles in oxidative metabolism (Barile et al., 2016). While other dietary-derived mono- or dual-ringed B vitamins, including vitamins B9 and B2, respectively (Eckle et al., 2014; Kjer-Nielsen et al., 2012; McInerney et al., 2024), or derivatives thereof can act as MR1 ligands, it has remained unclear whether the three-ringed RF molecule or catabolites thereof can directly bind MR1, and how this may relate to the presentation of microbial 5-A-RU derivatives. Recently, however, it was demonstrated that RF can indeed block MAIT cell responses to bacterial Ag presentation (Harriff et al., 2018; Kjer-Nielsen et al., 2012; Shibata et al., 2022); however, the molecular mechanism for this has been unclear. Moreover, when RF is catabolized, distinct structurally related products are formed, whereby FMF and lumichrome are the major by-products under neutral or acidic pH conditions, and both lumiflavin and lumichrome are formed under basic pH (Bitsch and Bitsch, 2016). Indeed, human blood contains RF catabolites (Barupal and Fiehn, 2019; Hardwick et al., 2004); however, the immunological roles of these molecules have not been explored. In this study, we provide cellular and structural evidence for direct binding of RF to MR1. Moreover, we show that catabolites of RF, including FMF, lumiflavin, and lumichrome, also bind MR1, extending the family of RF-derived MR1 binders beyond microbial 5-A-RU derivatives. Notably however, unlike previously described natural MR1 ligands, these RF catabolites blocked MR1-mediated Ag presentation inducing retention of MR1 in the ER.

The majority of MR1-binding ligands that have thus far been described are characterized by their unique ability to form a covalent Schiff base bond with Lys-43 of MR1 at the base of the A′-pocket (Awad et al., 2025a). This not only anchors the ligand to the Ag-binding groove but is thought to be an important feature of MR1 Ag presentation, whereby neutralization of Lys-43 in the ER results in egress of mature MR1 molecules to the cell surface for Ag presentation (McWilliam et al., 2016; McWilliam et al., 2020). How ligands that do not form a Schiff base fit into this paradigm remains unclear. The RF catabolites described here were incapable of Schiff base formation, and we observed that while they could bind ER-resident MR1, this MR1 did not egress from the ER, likely a result of the failure to neutralize Lys-43, and instead caused a retention of MR1 in the ER, which in turn drove downregulation of surface MR1. This is reminiscent of two previously described synthetic molecules, DB28 and NV18.1, neither of which bound MR1 covalently, and both of which drove MR1 surface downregulation (Salio et al., 2020). While in some instances, noncovalently bound ligands may be presented on the cell surface, e.g., via surface exchange, our data support a mechanism whereby the balance of ER-resident and surface-expressed MR1 is modulated by ligands that do or do not form the Schiff base interaction.

The biological consequences of this balance are likely a fine-tuning of MR1 Ag presentation. In our study, we show that RF catabolites can inhibit activation of human MAIT cells in response to 5-A-RU derivatives and this is driven by this mechanism of ER retention. Although the concentrations required to achieve this inhibition in our model *in vitro* system are relatively high, it is notable that RF and lumichrome can be detected in human serum in nanomolar quantities (Hardwick et al., 2004), and while the precise cellular concentrations of microbial metabolites required to activate MAIT cells are unknown, it is possible that under certain intracellular physiological settings, these catabolites or related molecules may indeed interfere with Ag presentation, thereby dampening MAIT cell responses. Similarly, while 5-OP-RU is a highly potent MAIT cell agonist, responses to lower affinity agonists for MAIT or other MR1-restricted T cells, such as those that recognize nucleobase adducts (Chancellor et al., 2025; Vacchini et al., 2024), may be more readily modulated. Indeed, RF and its transporters are known to be overexpressed in some tumors (Bartmann et al., 2019), and it is tempting to speculate that these RF catabolites may interfere with presentation of tumor-associated ligands such as M_3_Ade (Chancellor et al., 2025; Vacchini et al., 2024) as a mechanism of immune evasion. Moreover, the absence of the ribityl tail in the RF catabolites, and the stabilization with a smaller number of hydrogen bonds together with the lack of a Schiff base in comparison with 5-OP-RU, resulted in IC_50_ in the micromolar range as revealed by the fluorescence polarization assay. This weak binding relative to 5-OP-RU is in line with these other previously published host-derived ligands such as nucleobase adducts (Chancellor et al., 2025; Vacchini et al., 2024), bile acid derivatives (Ito et al., 2024), and vitamin B6 derivatives (McInerney et al., 2024), and further aligns with the potential of RF catabolites to modulate recognition of distinct Ags to varying degrees. Thus, the biological consequences of MR1 retention versus egress are likely a result of the balance of the relative bioavailability of MR1-binding ligands of varying binding capacities. Indeed, while in this manuscript we have assessed these ligands in isolation, a novel concept in MR1 biology emerges whereby a pool of weaker, ER-retaining ligands could potentially collectively serve to suppress or modulate MR1-restricted T cell responses, and while the concentration of one ligand in isolation may not be sufficient to inhibit strong Ags such as 5-OP-RU, the combined effect of multiple weaker ligands may fine-tune these T cell responses.

This study also showed that RF itself can bind MR1 but does not induce MR1 ER retention. Unlike the RF catabolites, RF neither sponsored adequate refolding with recombinant MR1 in solution nor changed the levels of intracellular Endo H–resistant MR1. Thus, RF itself does not seem to increase the intracellular folded MR1 to a significant level and is likely not occupying intracellular MR1, in contrast to its catabolites. In contrast, 100 μM RF induced a modest increase in MR1 surface expression in C1R.MR1 cells, and this is in line with previous work suggesting that the presence of empty MR1 molecules on the cell surface may be necessary for binding extracellular ligands, which cannot penetrate the ER or endosomes (McWilliam et al., 2016; McWilliam et al., 2020). Thus, we consider that RF itself, although capable of binding MR1 in a manner that supports surface exchange and crystallization of recombinant protein, likely does not bind ER-resident MR1 to induce ER retention. Whether this is a result of limited access to the ER or otherwise remains unclear. Furthermore, despite having a ribityl tail and acquiring the same orientation as 5-OP-RU in the MR1 pocket, RF fails to activate MAIT cells, likely for similar reasons.

We previously showed that the inherent stability of a ligand, together with its capability to form a Schiff base adduct with MR1, modulates the Ag’s capability to upregulate MR1 on the cell surface. In contrast, here we found that lumichrome was anchored to MR1 via a flavin bond with MR1-Lys43. Although lumiflavin and FMF have a very similar chemical structure, they did not form this flavin bond and exhibited markedly different binding modes and contacts within the MR1 A′-pocket, with their isoalloxazine rings rotated ∼90° relative to lumichrome and positioned closer to the MR1 α1 helix compared with the lumichrome, highlighting the malleability in the MR1 A′ and its ability to accommodate related molecules in highly distinct orientations. This unexpected interaction formed between lumichrome and MR1-Lys43 is an example of “covalent flavination,” a broadly reported process, in particular in a large number of flavoenzymes that use a covalently protein-bound flavin cofactor (Heuts et al., 2009). Here, this covalent tethering is a posttranslational and self-catalytic process that was shown to tune the reactivity of the flavin group so that it can fulfill its catalytic role in the flavoproteins (Starbird et al., 2017; Wang et al., 2022b). Approximately 1% of all known proteins are flavoproteins (Piano et al., 2017) where about 10% of these flavoproteins contain a covalently bound flavin (Heuts et al., 2009). Being ubiquitous in all domains of life, they have extremely versatile catalysis activity in a broad range of essential biochemical processes, including natural product biosynthesis, photosynthesis, DNA damage repair, chromatin modification, and immune-related activities (Piano et al., 2017; Teufel, 2017). Various immune-related proteins are covalently “flavinated” where the attached flavin group(s) appear to be important for their function (McNeil et al., 2014; Moreno et al., 2022; Pedrolli and Mack, 2014; Zafred et al., 2013; Zhong et al., 2020). From a structural perspective, there are nine known types of flavin–protein linkages up to date, comprising His, Tyr, Cys, Asp, Ser, or Thr attached to either FAD, FMN, or lumichrome (Wang et al., 2022b). In these linkages, the amino acid residue is attached to C6 or C8 of the flavin isoalloxazine ring or to the phosphate group. Here, we define a previously uncharacterized form of flavination in a nonenzymatic protein comprising the Lys-43 of MR1 covalently linked to C7 of the isoalloxazine ring. What role this has in MR1-mediated immunity is unclear. Notably, lumichrome binding does not induce ER egress but rather ER retention and thus is distinguished from those ligands that form a Schiff base. How these two distinct covalent bonds result in such different biological outcomes is unclear, and the range of natural molecules that can support covalent flavination with MR1 warrants further investigation.

In conclusion, we report lumichrome, among other RF catabolites, as a host-derived metabolite that weakly binds MR1, specifically reduces the cell surface MR1, and competes with MAIT agonists 5-OP-RU and RL-7-Me for MAIT cell activation, thereby modulating the MR1–MAIT cell axis. This may represent a natural suppression mechanism to regulate cell surface MR1 and thus avoid inadvertent immune activation or drive immune suppression.

## Materials and methods

### Ligands

RF (Cat. No. R9504), lumichrome (Cat. No. 103217), and alloxazine (Cat. No. A28651) were purchased from Sigma-Aldrich. Lumiflavin was supplied by Cayman Chemical (Cat. No. 20645). Ac-6-FP (Cat. No. 11.418) and 6-FP (Cat. No. 11.415) were synthesized by Schircks Laboratories. JYM20, 5A-RU, 5-OP-RU, and RL-7-Me were synthesized as previously described (Wang et al., 2022a; Awad et al., 2020a). All the ligands were dissolved in 0.1 M NaOH up to a concentration of 10 mM and diluted when required. The solubilized ligands were always protected from lab light during storage and experiments.

To synthesize FMF, a solution of sodium periodate (2.2 *g*, 10 mmol) in water (23.5 ml) was added to a suspension of RF (1.0 *g*, 2.7 mmol) in aqueous sulfuric acid (2 N, 26.5 ml) at 0°C. After stirring at the same temperature for 0.5 h, the mixture was warmed to room temperature. After stirring at the same temperature for a further 16 h, the mixture was adjusted to pH 3.9 (as measured by a pH meter) with solid sodium carbonate. The precipitate was collected by filtration and then washed successively with cold water, ethanol, and then diethyl ether. The precipitate was dried on high vacuum to give FMF as an orange solid (650 mg, 2.29 mmol, 85 %) as a mixture of the aldehyde and its corresponding hydrate. The NMR spectrum of FMF matched that reported in the literature (Crielaard et al., 2022).

### Screening of the binding affinity of the ligands to MR1 using fluorescence polarization assay

Fluorescence polarization–based cell-free assay has been recently developed for quantitating the relative binding affinities of putative ligands for MR1, including both activators and inhibitors of MAIT cells (Wang et al., 2022a). This assay reflects the inverse correlation between the IC_50_ of a given ligand and the binding affinity of that ligand, the ability to form a Schiff base, and the number of MR1/ligand noncovalent interactions. Various concentrations of RF and its catabolites were incubated in competition with 10 nM JYM20 (TAMRA fluorophore-conjugated weak MR1 ligand) for binding 100 nM empty hMR1 protein in fluorescence polarization assay buffer (25 mM HEPES, pH 7.5, 150 mM NaCl, 5 mM EDTA) (Wang et al., 2022a). The fluorescence polarization of TAMRA was measured after 24 of incubation at 37°C using PHERAstar microplate reader (BMG LABTECH). The ligand-binding curves were simulated by nonlinear regression with Prism software (GraphPad Software Inc.) using a sigmoidal dose–response curve. Here, the IC_50_ values reflect the binding affinity and are calculated as the ligand concentration required for 50% inhibition of JYM20 binding to MR1 molecules. The relative binding values (%) were then calculated as the percentage ratio between IC_50_ value of the substituted JYM20 and the IC_50_ value of the nonsubstituted ligand at the 10 nM concentration.

### Quantification of cell surface MR1

The level of MR1 expressed on the cell surface was measured after the exposure to the investigated ligands as previously described (Awad et al., 2020a; Wang et al., 2022a). C1R Ag-presenting B lymphocytes overexpressing MR1*01 (C1R.MR1), MR1^K43A^ mutant (C1R.MR1^K43A^), MR1^R9H^ mutant (C1R.MR1^R9H^), or HLA-A*02:01 (C1R-A2), in addition to the monocytic THP-1-MR1 cells, were used in this study. 2 × 10^5^ cells were incubated with RF catabolites for 3 or 16 h at 37°C and 5% CO_2_ in 200 μl folate-free RPMI 1640 medium from Gibco (Cat. No. 11875-093) fortified with 10% fetal bovine serum, sodium pyruvate (1 mmol/L), HEPES buffer (15 mmol/L), pH 7.2–7.5, 2% penicillin (100 U/ml), streptomycin (100 mg/ml), GlutaMAX (2 mmol/L), nonessential amino acids (0.1 mmol/L) (all from Thermo Fisher Scientific, Life Technologies), and 2-mercaptoethanol (50 mmol/L, Sigma-Aldrich) (RF-10). After incubation, the cells were first stained with Zombie Aqua Fixable Viability Kit from BioLegend (Cat. No. 423102) and then incubated with biotinylated 8F2F9 αMR1 antibody for 30 min on ice. The unbound antibody was then washed off with 2% fetal bovine serum/PBS (called 2% FACS hereafter) buffer. The cells were finally incubated with PE-conjugated streptavidin (30 μg/ml; BioLegend) and then washed with 2% FACS buffer. The PE fluorescence intensity reflects the surface MR1 level. The data were acquired with LSRFortessa X-20 (BD Biosciences) and Diva software (BD Biosciences) and analyzed using FlowJo software (BD Biosciences). The gating strategy is shown in Fig. S2 A.

### MAIT cell activation assay

C1R.MR1 cells (1 × 10^5^) were initially stained with CTV (Thermo Fisher Scientific) for 15 min and then incubated with RF catabolites for 20 h. SKW-3 cells (1 × 10^5^) overexpressing MAIT or MR1T TCRs were then tested for activation by coincubation at a 1:1 ratio with the CIR.MR1 cells for further 20 h in 200 μl complete medium with various RF catabolites, in the presence or absence of 1 nM 5-OP-RU or 100 nM RL-7-Me. Cells were subsequently stained with PE-Cy7-conjugated anti-CD69 (1:100; BD Biosciences) and PE-labeled anti-MR1 (1:100, clone 26.5; BD Biosciences), before analysis on a LSRFortessa (BD Biosciences) flow cytometer. Activation of the SKW-3 cells was reflected by the cell surface CD69 expression. The gating strategy is shown in Fig. S2 B.

### Human PBMCs

Human PBMCs were isolated from buffy coats isolated from healthy blood donors in Melbourne, Australia, and supplied by the Australian Red Cross after informed consent (agreement numbers: 17-08-VIC-16, 18-08-VIC-12, 20-10VIC-14, 22-11VIC-04, and 24-10VIC-15) in accordance with human ethics approval from the University of Melbourne Human Ethics Committee (ethics number: 30058). Whole blood was collected in heparin-coated tubes and centrifuged to separate the cellular fraction and plasma using lymphocyte separation solution (d = 1.077) (Nacalai Tesque). For the proliferation assay, PBMCs were labeled with CTV (Thermo Fisher Scientific) according to the manufacturer’s instructions. PBMCs were cultured with 5-OP-RU or RF catabolites in RPMI 1640 (Sigma-Aldrich) medium. Seven days after stimulation, PBMCs were analyzed by flow cytometry (Attune NxT flow cytometer, Thermo Fisher Scientific). For primary MAIT activation assays, isolated PBMCs were incubated with RF catabolites for 16 h in the presence or absence of 5-OP-RU, 5-A-RU, or zoledronate. To test the TCR-independent activation, PBMCs were preincubated with RF catabolites for 16 h followed by coincubation with PMA and ionomycin to a final concentration of 10 ng ml^−1^ and 1 μg ml^−1^ or coincubation with 5 μl of Dynabeads Human T Cell Activator CD3/CD28 (Thermo Fisher Scientific) for further 3 h. For surface staining, cells were then stained with anti-human CD69 (1:200; BD Biosciences), anti-human CD14 (1:100; BD Biosciences), anti-human CD19 (1:100; BD Biosciences), anti-human CD3 (1:100; BD Biosciences), anti-human TCR Vα7.2 (1:200; BioLegend), anti-human TCR Vδ2 (1:400; BD Biosciences), and the near-infrared viability dye for 30 min at room temperature. Following 2 washes with 2% FACS buffer, cells were incubated with MR1-5-OP-RU tetramers to a final concentration of 1 µg/ml for 30 min before analysis with a LSRFortessa (BD Biosciences) flow cytometer. For intracellular staining, PBMCs were incubated with the ligands for 1 h and then treated with GolgiPlug Protein Transport Inhibitor (1:1,000; BD Biosciences) overnight. On the following day, PBMCs were initially stained for the surface markers and then fixed and permeabilized using Cytofix/Cytoperm Fixation/Permeabilization Kit (BD Biosciences) for 30 min at 4°C. Anti-human TNF (1:200; BD Biosciences) was then added for 40 min at 4°C before washing and flow cytometry. The gating strategy is shown in Fig. S4 A.

### Western blot analysis of intracellular MR1

C1R.MR1 cells were cultured with different RF catabolites for 16 h, at 37°C. Samples were then treated with or without Endo H for 1-h incubation at 37°C. Cells were lysed, and lysate protein concentration was quantified by BCA assay. Equal amounts of samples (40 µg total protein) were separated on SDS-PAGE gels before being transferred onto nitrocellulose membranes and incubated with primary (anti-MR1; clone 8G3) (McWilliam et al., 2020) then corresponding secondary antibody (IRDye 800CW anti-mouse IgG, Cat. No. 925-32210; LICORbio). The protein bands were visualized by the Li-Cor system.

### Recombinant expression and purification of MR1 and the A-F7 MAIT TCR

*Escherichia coli* BL21(DE3) cells were transformed with plasmids encoding for the extracellular domains of MR1, β2m, and the AF-7 TCRα and TCRβ chains (Kjer-Nielsen et al., 2012; Patel et al., 2013; Wang et al., 2022a). The proteins were then expressed, and the inclusion bodies were purified, cleaned, and used for refolding. For both MR1 and AF-7 TCR, inclusion bodies were refolded through rapid dilution overnight as previously mentioned (Keller et al., 2017). To refold MR1 with ligands, 58 mg MR1 inclusion bodies along with 30 mg of β2m inclusion bodies were placed in 500 ml of refold buffer consisting of 0.1 M Tris, pH 8.0, 5 M of urea, 2 mM EDTA, 2.5 mM oxidized glutathione, 20 mM reduced glutathione, and 0.4 M of L-arginine for 16 h at 4°C. Ligands were dissolved in dilute NaOH and added directly into the refolding buffer. For photodegraded RF, 60 mg RF powder was dissolved in 1 L refolding buffer and then exposed to UV light through a UVA lamp for 30 min prior to the addition of inclusion bodies. The refolded MR1–ligand complex was dialyzed against 10 mM Tris, pH 8.0, overnight and purified using size-exclusion (Superdex 200, GE Healthcare) and anion-exchange (HiTrapQ, GE Healthcare) chromatography techniques (Awad et al., 2020a).

### Thermal stability assay

To investigate the stability of the MR1–RF catabolite complexes, the thermal shift assay was performed. SYPRO Orange (Sigma-Aldrich) was used as a fluorescent dye to monitor the protein unfolding upon heating. This assay was performed in a real-time detection system (Corbett RotorGene 3000). Each MR1-Ag complex was prepared in 10 mM Tris-HCl (pH 8) and 150 mM NaCl and heated from 28 to 95°C with a heating rate of 1°C min^–1^. The fluorescence intensity was measured (excitation at 530 nm and emission at 610 nm), and the unfolding process was followed in real time. Tm50 represents the temperature at which 50% of the protein was unfolded. All the experiments were performed in triplicates, at three independent times.

### Sample preparation and analysis by liquid chromatography-mass spectrometry (LC-MS)

Refolded MR1 samples containing ∼10 µg of refolded protein were treated 3:1 with acetonitrile containing 0.1% formic acid. These samples were vortexed and then allowed to stand at room temperature, and protected from light for 10 min, before centrifugation at 15,000 rcf for 3 min to pellet precipitated protein material. The supernatant was transferred to fresh lo-bind Eppendorf tubes and evaporated off using a centrifugal evaporator set to 38°C, reaching dryness after ∼1 h. The tubes were then reconstituted into an LC-MS–matched solvent (mobile phase A; 0.1% formic acid, 2% ACN in Optima Water). They were allowed to mix at 37°C for 30 min, prior to another centrifugation step at 15,000 rcf for 3 min, and the supernatant was transferred to LC-MS vials for analysis. Solvent standards for all analytes of interest were also prepared by dilution into the same mobile phase solvent (10 µM) from a 10 mM DMSO stock.

Samples were loaded into an Eksigent NanoLC system, which directly injected 5 μl of each sample into a Luna Omega 2 um Polar C18 (100A, LC column 50 × 0.3 mm). Gradient chromatography was used with mobile phase B (0.1% formic acid in 80% acetonitrile/Optima Water) ramping from 3% to 95% from 2 to 9 min, held for 2 min, and returned from 95 to 3% from 11 to 12 min, followed by a 3-min re-equilibration period (15 min total). MS data were acquired using a SCIEX 6600 TripleTOF, scanning MS1 from 100 to 700 m/z, and information-dependent acquisition (IDA) mode triggering MS2 data acquisition for ions exceeding 100 cps. Dynamic background subtraction and dynamic accumulation functions were used, and up to 10 MS2 scans were acquired per method cycle. Samples were run in both positive and negative modes, with a positive mode usually providing meaningful data.

### Crystallization and solving the structures of MR1*01–ligand complexes using MAIT TCR as a crystallization aid

The purified MR1–lumichrome complex was mixed with AF-7 TCR at 1:1 M ratio and incubated for 1 h on ice (Awad et al., 2020a). Because the other RF catabolites could not assist the refolding of MR1 to a reasonable amount in solution, we applied the established MR1 ligand displacement protocol to determine the structure of MR1 loaded with RF, FMF, and lumiflavin (Keller et al., 2017; Salio et al., 2020). Here, purified MR1-empty was concentrated and mixed with AF-7 TCR to a final concentration of 5 mg/ml and the ligand was diluted in this mixture as 1:10 (ligand: MR1) molar ratio. Empty MR1 was prepared as previously described (Keller et al., 2017). The ternary complexes were crystallized in 100 mM Bis-Tris propane (pH 6.1–6.5), 12–18% wt/vol PEG3350, and 200 mM sodium acetate using the hanging drop method. Complex crystals of AF-7 TCR–MR1–lumichrome and MR1–photodegraded RF complexes were formed within a week. After growth, the crystals were washed in mother liquor supplemented with 12% (vol/vol) glycerol and flash-frozen in liquid nitrogen. The x-ray diffraction from the ternary crystals was measured at the Australian Synchrotron (McPhillips et al., 2002), and the data are accessed through a Monash local account. The locally processed data were accessed via FileZilla and integrated using the XDS package (Kabsch, 2010). We solved the ternary structures at ∼ 1.9–2.2 Å resolution. The AF-7 TCR–MR1–ligand crystal structure was determined by molecular replacement using Phaser (McCoy et al., 2007) and AF-7 TCR-MR1-5-OP-RU structure (PDB: 6PUC) after removing 5-OP-RU. The structures were initially refined with Phenix.Refine (Liebschner et al., 2019), and then, the iterative model was built and improved in Coot (Emsley and Cowtan, 2004). In all structures, the cutoff for hydrogen bonds, salt bridges, and VDW interactions was set at 3.5, 4, and 4 Å, respectively. The quality of the structures was confirmed at the Research Collaboratory for Structural Bioinformatics Protein Data Bank Data Validation and Deposition Services website. All presentations of molecular graphics and figures were created with the PyMOL Molecular Graphics System, Version 2.0, Schrödinger, LLC.

### Statistical analysis

An unpaired two-tailed Student’s *t* test or one-way ANOVA with the Dunnett’s multiple comparison test was performed for the statistical analyses using GraphPad Prism (Version 9.1.0, GraphPad Software, Inc.), using either DMSO or NaOH as a control. Unless otherwise indicated, “ns” refers to “not significant,” and asterisks denote the level of statistical significance (*P < 0.05, **P < 0.01, ***P < 0.005, ****P < 0.001). P values were adjusted for multiple comparisons using GraphPad Prism version 9.1.0.

### Online supplemental material

Fig. S1 shows that RF catabolites refold with MR1 *in vitro*. Fig. S2 shows the gating strategies used in the SKW-3 cell line flow cytometry experiments. Fig. S3 shows the impact of RF catabolites on the activation of SKW-3 cell lines expressing different MR1-dependent TCRs. Fig. S4. shows the gating strategy used in PBMC flow cytometry experiments. Fig. S5 shows a mechanistic comparison between the flavin bond and Schiff base formation.

## Supplementary Material

Table S1 shows data collection and refinement statistics.

SourceData F3 is the source file for Fig. 3.

SourceData FS1 is the source file for Fig. S1.

## Data Availability

The data underlying Figs. 1, 2, 3, 4, and 5; and Figs. S1, S2, S3, S4, and S5 are available in the published article and its online supplemental material, respectively. The atomic coordinates of AF-7 TCR-MR1 in complex with RF, FMF, lumiflavin, and lumichrome have been deposited in the Protein Data Bank (https://www.rcsb.org) under accession codes 9O05, 9O06, 9O07, and 9O08, respectively.
