# Supplementary material for: The antigen-presenting molecule MR1 binds host-generated riboflavin catabolites
Source: J Exp Med. 2025 Nov 26;223(2):e20250711. doi: 10.1084/jem.20250711 (PMC12650265; doi:10.1084/jem.20250711)
Supplement: Table S1 — shows data collection and refinement statistics. [file jem_20250711_tables1.docx]

**Table S1. Data collection and refinement statistics.**

|  | **A-F7 TCR-MR1-Lumichrome** | **A-F7 TCR-MR1-Riboflavin** | **A-F7 TCR-MR1-Lumiflavin** | **A-F7 TCR-MR1-FMF** |
| --- | --- | --- | --- | --- |
| **Resolution range** | 45.43 - 2.2 (2.22 - 2.20) | 47.01 - 1.95 (1.97 - 1.95) | 49.32 - 1.97 (1.99 - 1.97) | 48.16 - 2.0 (2.02 - 2.00) |
| **Space group** | C 1 2 1 | C 1 2 1 | C 1 2 1 | C 1 2 1 |
| **Unit cell**  **a, b, c (Å)**  **α, β, γ (°)** | 216.43 69.73 143.13  90 104.38 90 | 217.38 70.77 143.63  90 104.70 90 | 212.71 69.29 141.17 90 103.48 90 | 217.42 70.32 143.28  90 104.63 90 |
| **Total reflections** | 204792 (6827) | 295931 (9789) | 278045 (9097) | 279863 (9326) |
| **Unique reflections** | 104865 (3501) | 152073 (5066) | 141041 (4650) | 141294 (4721) |
| **Multiplicity** | 2.0 (2.0) | 1.9 (1.9) | 2.0 (2.0) | 2.0 (2.0) |
| **Completeness (%)** | 99.45 (99.37) | 98.67 (98.01) | 99.66 (99.51) | 99.52 (99.43) |
| **Mean I/sigma(I)** | 8.60 (1.58) | 14.68 (1.69) | 11.03 (1.70) | 10.29 (1.24) |
| **Wilson B-factor (Å^2^)** | 41.26 | 31.88 | 37.75 | 39.30 |
| **R-merge** | 0.04655 (0.4859) | 0.02797 (0.47) | 0.02892 (0.4591) | 0.02778 (0.4551) |
| **R-meas** | 0.06584 (0.6872) | 0.03956 (0.6647) | 0.04089 (0.6493) | 0.03929 (0.6437) |
| **R-pim** | 0.04655 (0.4859) | 0.02797 (0.47) | 0.02892 (0.4591) | 0.02778 (0.4551) |
| **CC1/2** | 0.997 (0.802) | 0.999 (0.743) | 0.999 (0.618) | 0.999 (0.838) |
| **CC*** | 0.999 (0.943) | 1 (0.923) | 1 (0.874) | 1 (0.955) |
| **R-work** | 0.1663 (0.2742) | 0.1660 (0.2737) | 0.1574 (0.2417) | 0.1739 (0.3418) |
| **R-free** | 0.2190 (0.3404) | 0.1657 (0.2979) | 0.1920 (0.2682) | 0.2114 (0.3838) |
| **Number of non-hydrogen atoms** | 14146 | 14765 | 14486 | 14166 |
| **macromolecules** | 13053 | 13043 | 13031 | 12969 |
| **ligands** | 67 | 100 | 45 | 110 |
| **solvent** | 1026 | 1622 | 1410 | 1087 |
| **Protein residues** | 1607 | 1606 | 1606 | 1606 |
| **RMS (bonds) (Å)** | 0.007 | 0.006 | 0.007 | 0.007 |
| **RMS (angles)** | 0.86 | 0.82 | 0.85 | 0.81 |
| **Ramachandran favored (%)** | 97.85 | 98.23 | 98.29 | 97.85 |
| **Ramachandran allowed (%)** | 2.09 | 1.71 | 1.71 | 2.15 |
| **Ramachandran outliers (%)** | 0.06 | 0.06 | 0.00 | 0.00 |
| **Rotamer outliers (%)** | 1.49 | 2.12 | 1.84 | 2.07 |
| **Average B-factor** | 48.17 | 42.86 | 42.98 | 53.82 |
| **macromolecules** | 48.00 | 42.31 | 42.48 | 53.83 |
| **ligands** | 46.29 | 37.96 | 32.09 | 45.23 |
| **solvent** | 50.43 | 47.63 | 47.97 | 54.52 |

*Statistics for the highest-resolution shell are shown in parentheses.
