# Supplementary figures and images for: The antigen-presenting molecule MR1 binds host-generated riboflavin catabolites
Source: J Exp Med. 2025 Nov 26;223(2):e20250711. doi: 10.1084/jem.20250711 (PMC12650265; doi:10.1084/jem.20250711)

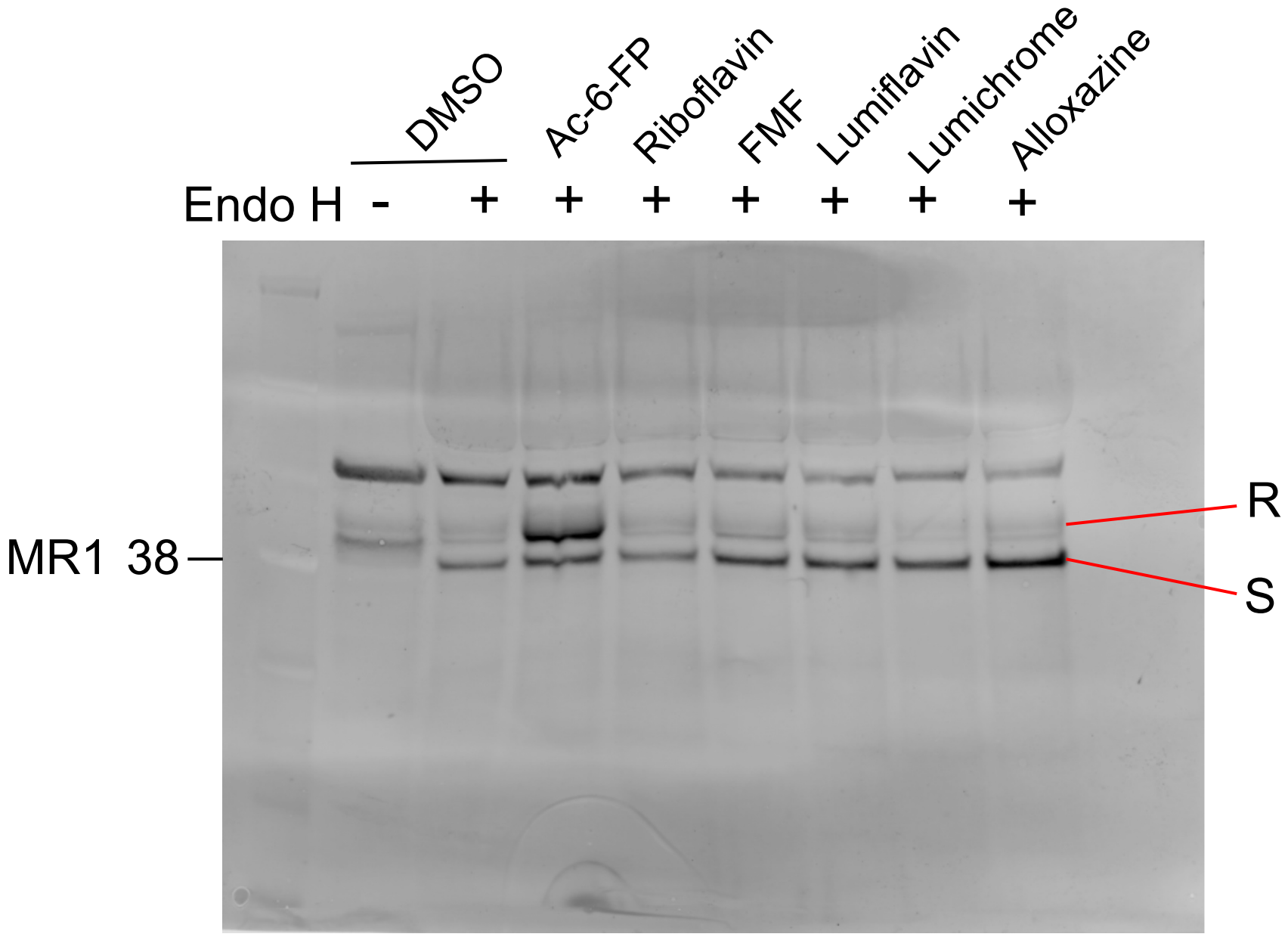

$\beta$ -actin

49—  
38—

| Endo H | DMSO |   | Ac-6-FP | Riboflavin | FMF | Lumiflavin | Lumichrome | Alloxazine |
|--------|------|---|---------|------------|-----|------------|------------|------------|
| -      | +    | + | +       | +          | +   | +          | +          | +          |

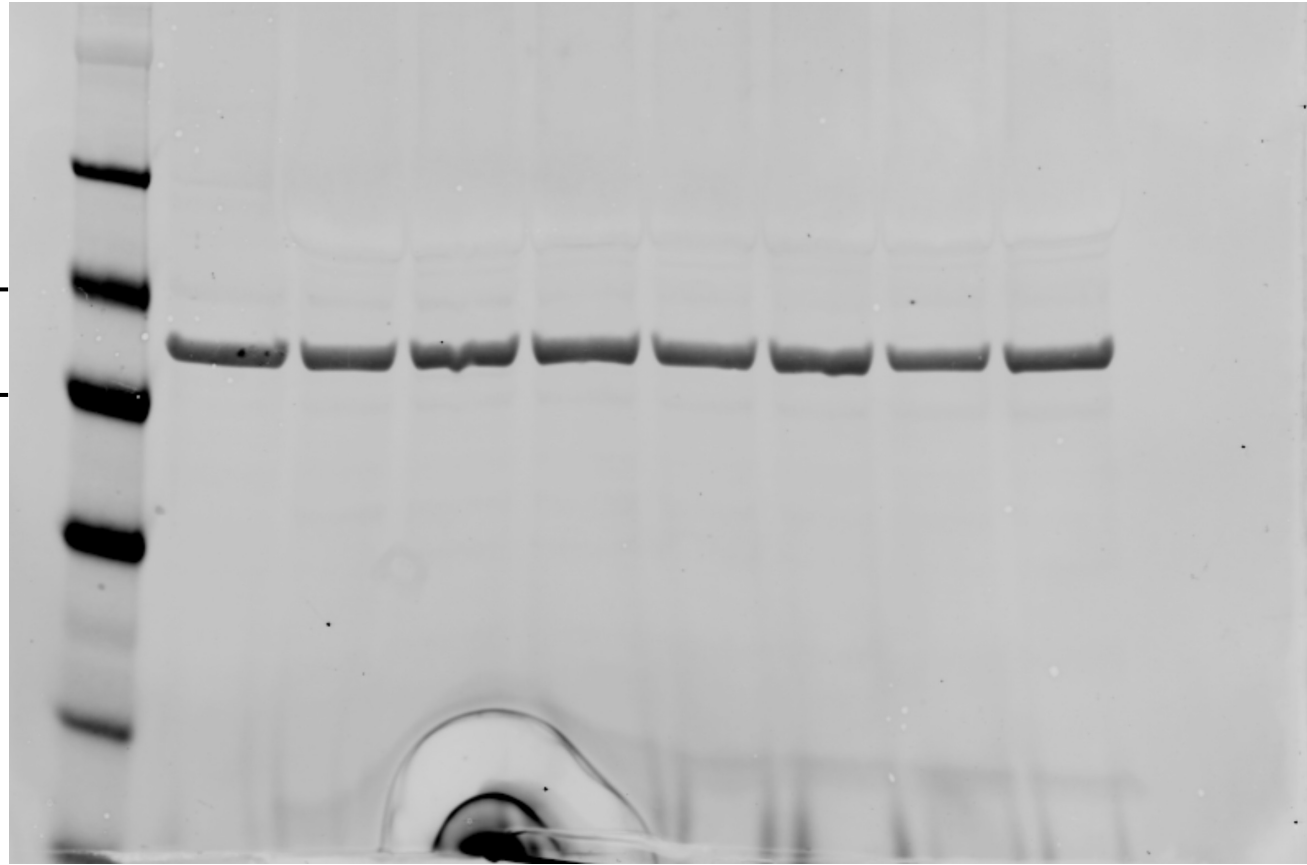

Supplement: SourceData F3 — is the source file for Fig. 3. [file jem_20250711_sourcedataf3.pdf]

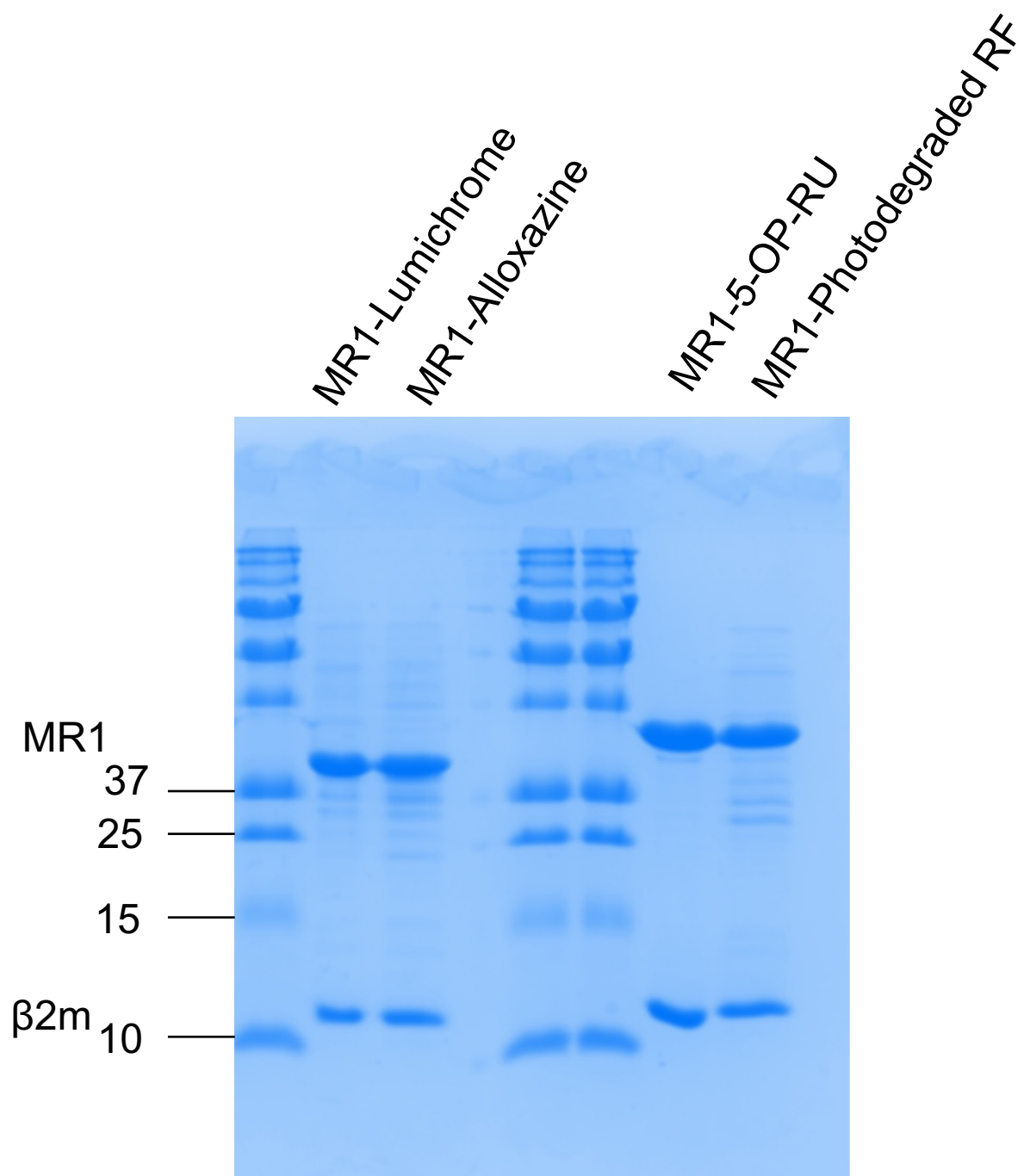

Supplement: SourceData FS1 — is the source file for Fig. S1. [file jem_20250711_sourcedatafs1.pdf]
